# Deglycosylation and truncation in the neuraminidase stalk are functionally equivalent in enhancing the pathogenicity of a high pathogenicity avian influenza virus in chickens

**DOI:** 10.1128/jvi.01478-24

**Published:** 2025-02-14

**Authors:** Daiki Kobayashi, Takahiro Hiono, Hiromu Arakawa, Hiroyuki Kaji, Ayako Ohkawara, Takaya Ichikawa, Hinako Ban, Norikazu Isoda, Yoshihiro Sakoda

**Affiliations:** 1 Laboratory of Microbiology, Department of Disease Control, Faculty of Veterinary Medicine, Hokkaido University 12810 , Sapporo, Hokkaido, Japan; 2 One Health Research Center, Hokkaido University 12810 , Sapporo, Hokkaido, Japan; 3 Institute for Vaccine Research and Development (HU-IVReD), Hokkaido University 12810 , Sapporo, Hokkaido, Japan; 4 International Collaboration Unit, International Institute for Zoonosis Control, Hokkaido University 12810 , Sapporo, Hokkaido, Japan; 5 Systems Biology Division, Institute for Glyco-core Research (iGCORE), Nagoya University 12965 , Nagoya, Japan; 6 Department of Microbiology and Immunology, Faculty of Medicine, Hokkaido University 12810 , Sapporo, Hokkaido, Japan; Emory University School of Medicine, Atlanta, Georgia, USA

**Keywords:** pathogenicity, high pathogenicity avian influenza virus, neuraminidase, *N*-glycosylation, site-specific glycan occupancy

## Abstract

Influenza A viruses with fewer amino acids in the neuraminidase (NA) stalk domain are primarily isolated from chickens rather than wild ducks, indicating that a shortened NA stalk is considered an adaptation marker of avian influenza viruses (AIVs) to chickens. Experimental passages of an H7N7 nonpathogenic AIV (rgVac2-P0) in chickens resulted in a highly pathogenic variant (Vac2-P3L4) with a 34-amino-acid deletion in the NA stalk, encompassing five potential *N*-glycosylation sites. To investigate how amino acid truncation and deglycosylation in the NA stalk contribute to increased pathogenicity, a virus with glycosylation-deficient mutations at these sites (rgVac2-P3L4/P0NAΔGlyco) was constructed. Contrary to expectations, chickens inoculated with rgVac2-P3L4/P0NAΔGlyco exhibited variable clinical outcomes, attributed to the genetic instability of the virus. A single mutation stabilized the virus, and the mutant (rgVac2-P3L4/P0NAΔGlyco-Y65H) resulted in higher pathogenicity compared with a virus with restored glycosylation (rgVac2-P3L4/P0NA-Y65H). Glycan occupancy analysis revealed 3–4 glycans at the five potential sites. In functional analysis, glycosylation-deficient mutants, similar to the short-stalk NA virus, showed significantly reduced erythrocyte elution activity. Additionally, mutational analysis indicated variable contributions of *N*-glycans to elution activity across the sites. Moreover, the functionally most contributing sites of the five potential *N*-glycosylation motifs were consistently included in the amino acid deletions of the stalk-truncated NA in N7-subtyped field isolates, despite the varying truncation position or length. These findings suggest that the loss of glycosylation is functionally equivalent to a reduction in amino acids, and it plays a crucial role in enhancing pathogenicity in chickens and affecting NA function.

**IMPORTANCE:**

Avian influenza poses significant economic challenges to the poultry industry and presents potential risks to human health. Understanding the molecular mechanisms that facilitate the emergence of chicken-adapted avian influenza viruses (AIVs) from non-pathogenic duck-origin influenza viruses is crucial for improving AIV monitoring systems in poultry and controlling this disease. Amino acid deletions in the neuraminidase (NA) stalk domain serve as one of the molecular markers for AIV adaptation to Galliformes. This study highlights the critical role of *N*-glycosylation in the NA stalk domain in the pathogenesis of high pathogenicity avian influenza viruses in chickens. The findings propose a novel theory that the loss of glycosylation at the NA stalk domain, rather than a reduction in stalk length, is responsible for both NA function and increased virus pathogenicity in chickens.

## INTRODUCTION

Influenza A viruses, which belong to the family *Orthomyxoviridae* and the genus *Alphainfluenzavirus*, feature two surface glycoproteins: hemagglutinin (HA) and neuraminidase (NA) (1). HA binds to sialylated glycoconjugates with terminal sialic acid linked to penultimate galactose via α2,3 or α2,6 linkages, facilitating viral attachment to host cells (2). NA, a tetrameric type-II transmembrane protein, comprises four domains: a short cytoplasmic tail, a transmembrane region, a stalk, and a globular head (3). The NA possesses sialidase activity, and its catalytic site is located at the globular head domain. The sialidase activity of NA promotes the release of progeny virions and facilitates viral spread from cell to cell. Additionally, NA aids in viral entry into host cells by removing “decoy” receptors (4, 5).

Influenza A viruses are classified into 16 HA subtypes (H1–16) and 9 NA subtypes (N1–9) based on their antigenicity (6–8). All HA and NA subtypes have been isolated from water birds; therefore, they are considered the natural hosts of avian influenza viruses (AIVs) (6, 8, 9). AIVs circulating among water birds can be transmitted to chickens via domestic water and terrestrial birds, where they acquire pathogenicity through multiple replications within the chicken population (10). Previous studies have shown that viruses with truncated amino acids in the NA-stalk domain have been isolated from chickens and turkeys in the field (11–13) and have also been selected during laboratory adaptation of duck-derived viruses to other hosts (14–17). Viruses with short-stalk NA have been identified in seven NA subtypes, including N1–3, N5–7, and N9 (18–20). Notably, short-stalk NA has not been found in nonpathogenic viruses circulating in wild aquatic birds but is frequently observed in viruses circulating in chickens (11, 19). Viruses with short-stalk NA demonstrate biological advantages in chickens compared to those with full-stalk NA, including enhanced virus growth, pathogenicity, and transmissibility (21–23). Therefore, the short-stalk NA is considered a relic of molecular evolution, reflecting the adaptation of AIVs from wild aquatic birds to chickens.

Viruses with short-stalk NA exhibit reduced sialidase activity compared to those with full-stalk NA, but only when tested with bulky substrates like fetuin or erythrocytes (24). Both short-stalk and full-stalk NA viruses, however, show similar reactivity to smaller compounds, such as *N*-acetylneuraminyllactose or 2-(4-methylumbelliferyl)-α-D-*N*-acetylneuraminic acid (MUNANA) (21, 24). This pattern is often explained by the “limited access theory,” which posits that the short-stalk NA is partially obscured by the HA canopy, limiting its access to bulky sialylated substrates (24). However, Durrant et al. (25) challenged this theory, arguing that it does not fully account for the reduced sialidase activity of short-stalk NA. They noted that a 20-amino-acid deletion in the NA-stalk leaves approximately 81% of the HA height intact, suggesting that access to the NA globular head domain is unlikely to be significantly impeded by the reduction in stalk height. Furthermore, structural analyses using cryo-electron microscopy have shown that NA forms localized clusters rather than being evenly distributed across the virion surface (26). These clusters could potentially facilitate substrate access to the catalytic site of the short-stalk NA. Therefore, the impact of stalk deletion on NA biological function remains debatable.

To explore the molecular mechanisms by which duck-derived, nonpathogenic AIVs acquire high pathogenicity in chickens, several studies have conducted serial passages of AIVs in chickens (27–29). Maruyama et al. (27) and later Ichikawa et al. (30) performed serial passages of a genetically modified, nonpathogenic H7N7 AIV (rgVac2sub-P0) in chickens. This led to the emergence of a high pathogenicity avian influenza virus (HPAIV; Vac2sub-P3L4) with a 34-amino-acid truncation in the NA-stalk domain under functional coevolution of HA and NA. Their studies revealed that this NA-stalk deletion along with mutations in HA was a crucial adaptation for the virus to achieve a highly pathogenic phenotype in chickens when birds were challenged via the intranasal route, in addition to polybasic amino acids at the HA cleavage site. Notably, the deletion site in the NA-stalk domain included five potential *N*-glycosylation sites. Protein modification on the stem by *N*-glycans generally modulates the biophysical properties and stability of proteins by adding large hydrophilic carbohydrates (31). For example, deglycosylation of the HA stem region has been shown to reduce viral replication and pH stability (32). Consequently, it is hypothesized that the loss of *N*-glycosylations in the NA-stalk may influence NA biological function and virus pathogenicity in chickens. To test this hypothesis, the present study rescued a virus with a glycosylation-deficient NA-stalk and evaluated its pathogenicity in chickens. Despite initial genetic instability *in vivo*, a single amino acid substitution corrected this instability and resulted in increased pathogenicity compared with the virus with retained NA-stalk glycosylation. Additionally, viruses with glycosylation-deficient NA-stalk demonstrated decreased erythrocyte elution activity, similar to that of a virus with truncated NA-stalk.

## RESULTS

### Pathogenicity of a recombinant virus with glycosylation-deficient NA stalk in chickens

**Fig 1:**
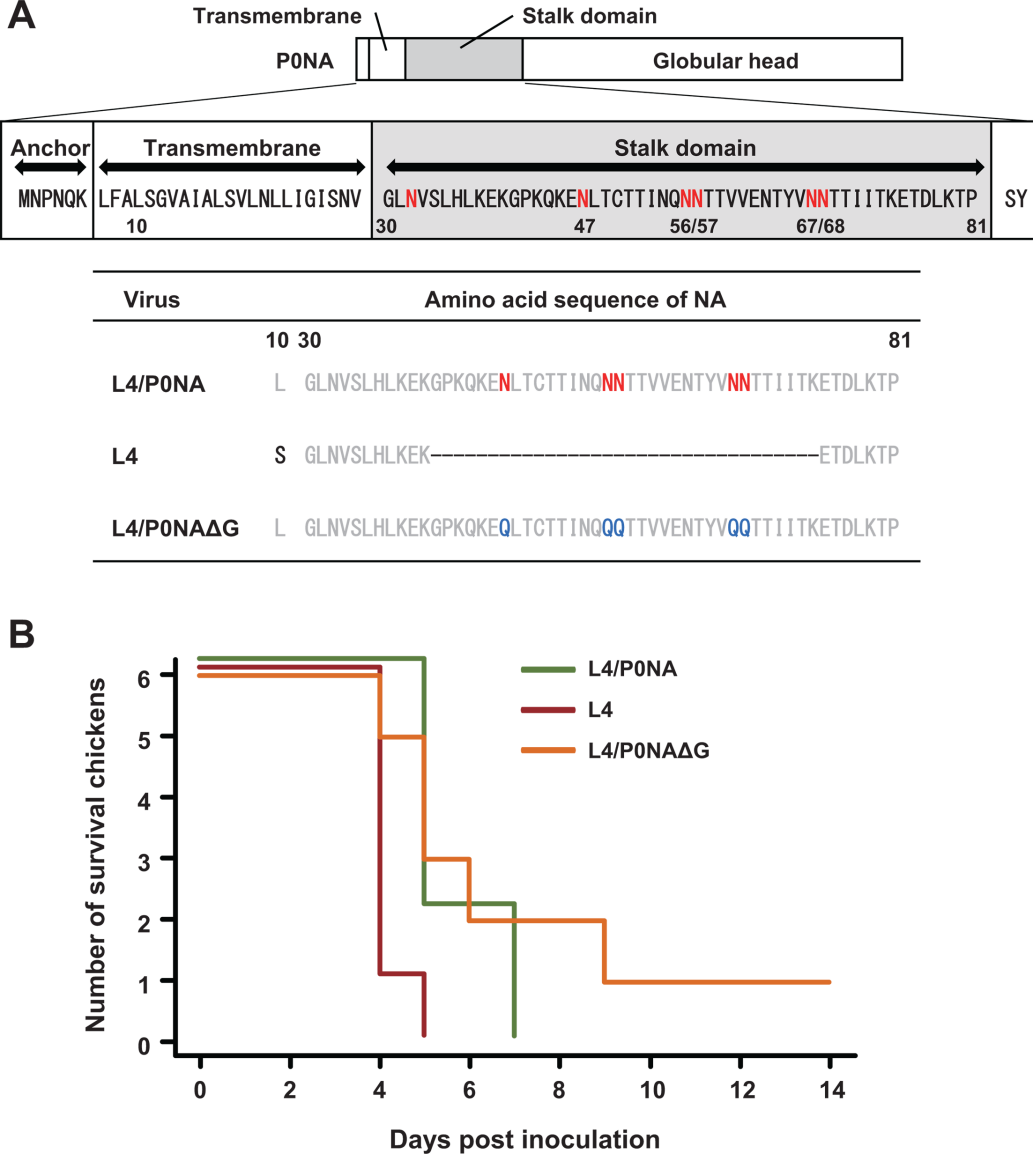
Survival of chickens intranasally inoculated with NA stalk mutant viruses. (**A**) Scheme of NA proteins of Vac2-P0NA, constructed from a membrane anchor, transmembrane domain, stalk domain, and globular head domains is shown. P0NA is a full-stalk NA, whereas P3L4NA, which was derived from several passages of rgVac2-P0 in chickens, has a 34-amino-acid deletion in the NA-stalk domain and one amino acid substitution in the transmembrane domain. P0NAΔGlyco was created by introducing N/Q mutations at the five potential *N*-glycosylation sites in the stalk domain. A series of viruses carrying either NA gene and accompanied by the L4 backbone were generated through reverse genetics in this study. (**B**) Six 6-week-old chickens were intranasally inoculated with 100 µL of each virus at 10^4.0^ TCID_50_ (50% tissue culture infectious dose) and observed for 14 days.

To verify the previous observation that truncation of the amino acids in the NA-stalk domain contributes to increased intranasal pathogenicity in chickens, a pair of viruses harboring a full-length NA stalk (rgVac2-P3L4/P0NA; L4/P0NA) and short NA stalk featuring a 34-amino-acid deletion (rgVac2-P3L4; L4) were used (Fig. 1A and Tables S1 and S2; [30]). Six 6-week-old chickens were intranasally inoculated with 10^4.0^ TCID_50_ of each virus, and clinical manifestations were monitored for 14 days. The viral pathogenicity in chickens showed high reproducibility with previous results: all chickens inoculated with L4 died within 4 days post-inoculation (dpi), whereas those inoculated with L4/P0NA exhibited delayed clinical onset and survived for 7 days (Fig. 1B). Thus, the truncation of the NA-stalk domain in L4 was proven to increase its pathogenicity in chickens.

Subsequently, to assess the functional significance of the loss of *N*-glycosylations on the stalk domain of NA, N/Q mutations that abrogate *N*-glycosylation sequons (N-X-S/T) were introduced into the NA-stalk domain of P0NA. A virus harboring this NA gene segment (P0NA∆Glyco) with the backbone of the other seven gene segments from L4 was generated (rgVac2-P3L4/P0NA∆Glyco; L4/P0NA∆G; Fig. 1A). The virus, L4/P0NA∆G, was intranasally inoculated into chickens to compare its pathogenicity. Interestingly, chickens inoculated with L4/P0NA∆G exhibited various clinical courses (Fig. 1B). One chicken died on 4 dpi, and three were euthanized on 4 or 5 dpi due to severe neurological signs. Conversely, another chicken died on 9 dpi, and one survived the 14-day observation period. Furthermore, the surviving chicken seroconverted during the experimental period (8 HI). To investigate the cause of the variable pathogenesis of L4/P0NA∆G, NA genes from viruses recovered from the infected chickens were sequenced. Amino acid substitutions or deletions in the NA-stalk domain were observed in all viruses recovered from chickens infected with L4/P0NA∆G (Table S3). Conversely, no mutations in the NA-stalk were found in viruses recovered from chickens inoculated with either L4/P0NA or L4, suggesting the genetic instability of P0NA∆Glyco. Although a total of four distinct amino acid mutants were identified among the five individuals, the Y65H mutation was found in viruses recovered from two co-housed chickens. Notably, one chicken (#34) showed severe neurological symptoms on 5 dpi, whereas the co-housed chicken (#33) began to exhibit mild clinical signs on 7 dpi. The observation was supported by whole genome sequencing and the subsequent population analysis of minor variants (Table S4). The population of Y65H mutant was higher in the brain (91.7%) and lung (90.0%) of chicken #33 compared with those of chicken #34 (61.2% in brain and 74.8% in lung). Additionally, a minor variant I621V in PA of chicken #34 (0.7% in brain and 0.9% in lung) turned out to be dominant in chicken #33 (88.4% in brain and 73.9% in lung). This suggests that the Y65H mutant was likely transmitted from one chicken (#34) to the other (#33) without further mutation in the NA-stalk. These results indicate that the Y65H mutant appears to be genetically more stable than the original L4/P0NA∆G in chickens.

### Genetic stability of Y65H mutant of L4/P0NAΔG

**Fig 2:**
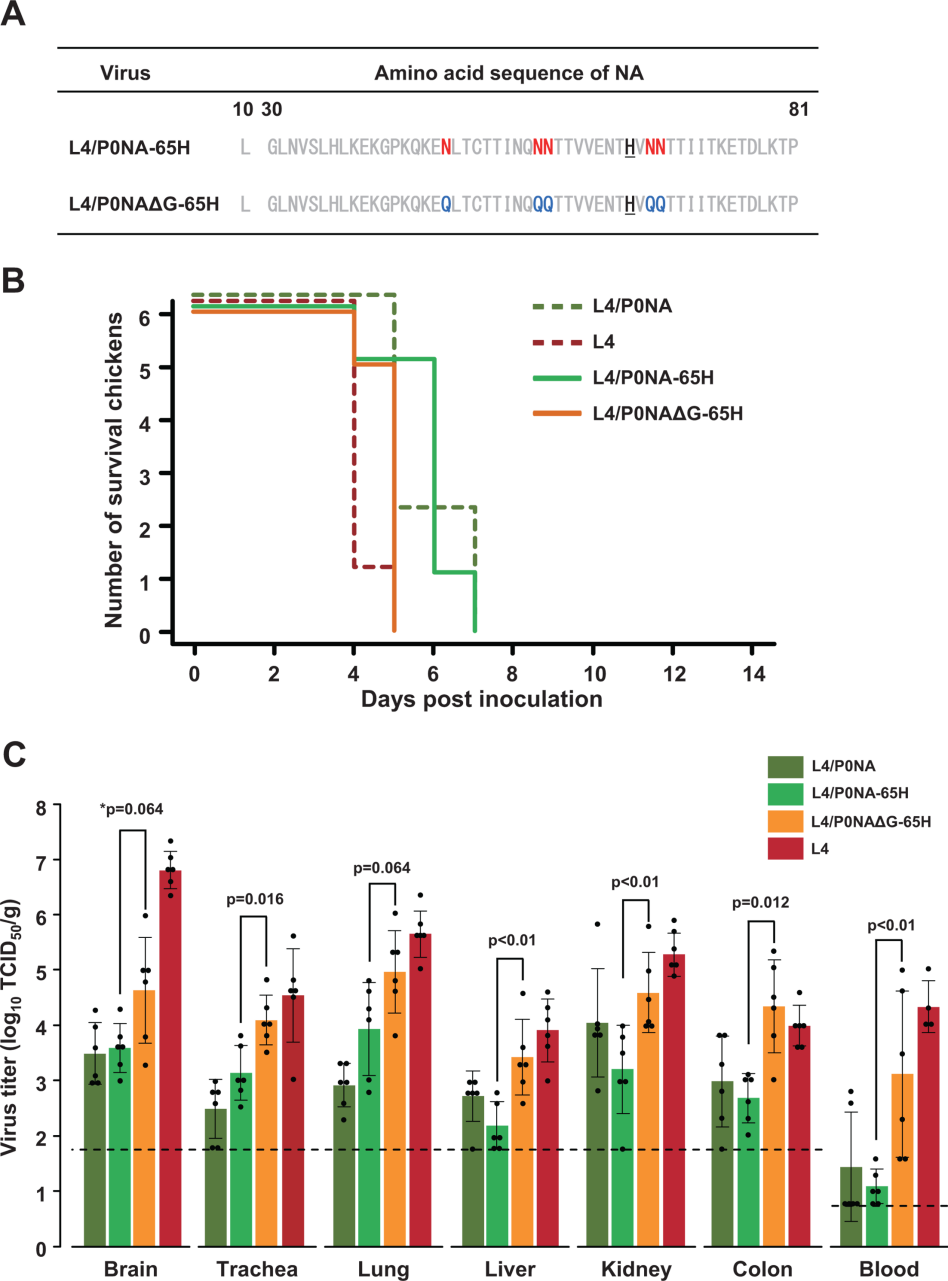
Survival of chickens and virus recovery from chickens intranasally inoculated with L4 mutants harboring Y65H substitution in the NA-stalk domain (**A**) Amino acid sequences of Vac2NA mutants with the Y65H mutation are shown. Recombinant viruses L4/P0NA-65H and L4/P0NAΔG-65H were generated using reverse genetics. (**B**) Six 6-week-old chickens were intranasally inoculated with 100 µL of each Y65H mutant at 10^4.0^ TCID_50_ and observed for 14 days. Survival of chickens inoculated with L4/P0NA and L4 is indicated with break lines. (**C**) Virus titers in the organs of chickens intranasally inoculated with each virus were evaluated 3 days post-inoculation. The dotted line indicates the threshold for virus titration (<1.8 log_10_ TCID_50_/g for organ samples and <0.8 log_10_ TCID_50_/g for blood). *Statistical analysis was performed using the Mann–Whitney *U* test between L4/P0NA-65H (light green) and L4/P0NAΔG-65H (orange). Virus titers below the detection limit were considered 1.8 log_10_ TCID_50_/g for tissue samples and 0.8 log_10_ TCID_50_/g for blood for statistical analysis.

To validate the genetic stability of the Y65H substitution in the NA protein of L4/P0NAΔG, L4/P0NAΔG and the Y65H mutants were serially passaged in embryonated chicken eggs. To achieve this, the Y65H substitution, changing thymidine to cytosine, was introduced into the NA-coding plasmid, and L4/P0NAΔG-65H was rescued via reverse genetics (Fig. 2A). Subsequently, two viruses—L4/P0NAΔG and L4/P0NAΔG-65H—were independently passaged three times in chicken embryos. Two distinct variants of L4/P0NAΔG were obtained (Table S5), whereas no mutants were obtained from the descendants of L4/P0NAΔG-65H. This indicates that the Y65H mutation in the NA-stalk seems to stabilize the genetic properties of P0NAΔGlyco.

To confirm the impact of the Y65H mutation on the genetic stability of the NA stalk-coding region, the RNA secondary structure was predicted through *in silico* analyses and compared. RNA sequences (129 nt) coding the NA-stalk region of P0NAΔGlyco formed a stem-loop structure consisting of 34 nucleotides, as predicted by both minimum free energy and centroid structures in RNAfold (33). Conversely, the stem-loop structure was abolished in the Y65H mutant due to a single substitution from uracil to cytosine (Fig. S1). The stem-loop structure was not observed in the RNA sequences of either P0NA or P0NA-Y65H. Predictions made using CentroidFold also suggested stem-loop formation in the stalk-coding region of P0NAΔGlyco (Fig. S2). Thus, the presence of the stem-loop structure in the NA-stalk region of P0NAΔGlyco appears to enhance the frequency of nucleotide substitutions or deletions by inducing viral polymerase slippage. Conversely, the Y65H mutation in P0NAΔGlyco compensates for the instability of the RNA secondary structure in the NA-stalk region. Accordingly, these results support the genetic stability of L4/P0NAΔG-65H.

### NA-Stalk deglycosylated virus with Y65H mutation (L4/P0NAΔG-65H) shows increased pathogenicity in chickens

The pathogenicity of a pair of genetically stable viruses, L4/P0NA-65H and L4/P0NAΔG-65H, was assessed in chickens to further investigate the functional significance of the loss of glycosylation in the NA-stalk. For this purpose, six 6-week-old chickens were challenged with 10^4.0^ TCID_50_ of each virus, and clinical manifestations were monitored. Chickens inoculated with L4/P0NA-65H exhibited similar clinical onsets to those inoculated with L4/P0NA, with no significant differences in the survival curve (*P* value adjusted using the Bonferroni method following pairwise comparisons using the log-rank test, *P* = 1.000; Fig. 2B), suggesting that the Y65H substitution in the NA-stalk did not affect viral pathogenicity in chickens. Conversely, all chickens inoculated with L4/P0NAΔG-65H developed severe neurological signs within 4 dpi and died within 5 days. The time required to affect all individuals inoculated with L4/P0NAΔG-65H was shorter than that for L4/P0NA-65H and no longer than that for L4. The difference in the survival curve was not statistically significant (*P* = 0.118 vs L4/P0NA-65H; *P* = 0.162 vs. L4, which was compared with *P* = 0.023 for L4/P0NA-65H vs L4). Notably, no mutations, including substitutions or deletions, were found in the viruses recovered from chickens inoculated with L4/P0NA-65H and L4/P0NAΔG-65H. Chickens were also euthanized on 3 dpi to investigate viral loads in blood and tissue samples. The virus titers in the trachea and lung of L4/P0NAΔG-65H-inoculated chickens were higher than those in chickens inoculated with L4/P0NA-65H (Fig. 2C). Additionally, higher levels of viremia were confirmed in the blood samples of chickens inoculated with L4/P0NAΔG-65H compared with those inoculated with L4/P0NA-65H. This resulted in higher viral loads in the brain, liver, kidney, and colon. These results demonstrate that the loss of potential *N*-glycosylation sites in the NA-stalk domain contributed to increased viral growth in primary target organs, such as the trachea and lungs, leading to higher viremia on 3 dpi and shortened survival periods for the infected chickens. When comparing L4/P0NAΔG-65H with L4, comparable virus loads were observed in each tissue sample except the brain (Fig. 2C). This finding supports the notion that the loss of glycosylation in the NA stalk is sufficient for rapid viral growth in chickens and suggests an additional advantage for viruses with short-stalk NA in replicating in the chicken brain.

### Loss of glycosylation in the NA stalk reduces elution activity of the virus virions against chicken erythrocytes

**Fig 3:**
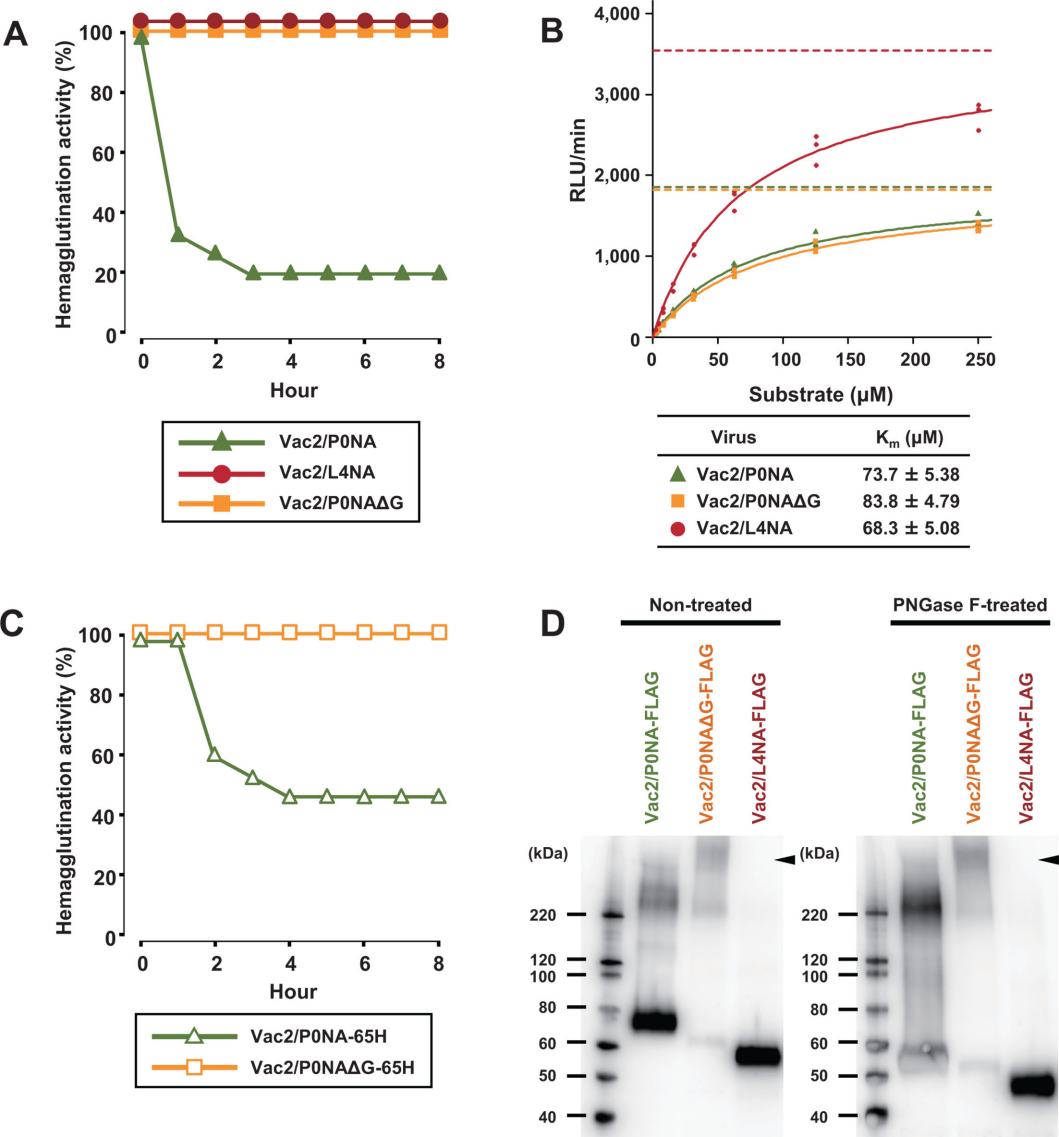
Biological characteristics of Vac2 mutants with or without glycosylation in the NA-stalk domain. (**A**) Erythrocyte elution activity of Vac2/P0NA, Vac2/L4NA, and Vac2/P0NAΔG was assessed. These mutants harbor full-stalk NA, short-stalk NA, and glycan-deficient-stalk NA, respectively. (**B**) Sialidase activity of Vac2/P0NA, Vac2/L4NA, and Vac2/P0NAΔG to MUNANA was assessed. The initial reaction rate at each condition was plotted to calculate the Michaelis constant, *K*_m_. The data were represented as the mean ± SEM of three independent experiments. (**C**) Erythrocyte elution activity of Vac2 mutants with partially glycosylated NA in the stalk was assessed. (**D**) Molecular weight of the NA proteins was compared to assess glycosylation of the NA-stalk domains under nontreated and PNGase F-treated conditions. Arrowhead indicates the aggregates of NA proteins of Vac2/P0NAΔG on the top of the resolving gel.

Because the deglycosylation of the NA-stalk domain in L4/P0NA-65H enhanced viral pathogenicity in chickens, the biological function of the NA-stalk was focused on. Previous studies have demonstrated that viruses with a short NA stalk have reduced elution activity against chicken erythrocytes, which is consistent with their higher pathogenicity compared with viruses with a full NA stalk (17, 30, 34). Thus, this study aimed to address the contribution of deglycosylation in the NA stalk to erythrocyte elution activity. Viruses with an L4 backbone could not achieve high HA titers (<2 HA) in eggs due to their exceptionally high lethality in chicken embryos. Therefore, a set of viruses—Vac2/P0NA, Vac2/L4NA, and Vac2/P0NA∆G—using the backbone of a nonpathogenic AIV (Vac2) were generated and used for the erythrocyte elution assay as previously described (Table S1; 34). As expected, the virus with full-stalk NA, Vac2/P0NA, efficiently eluted chicken erythrocytes, whereas the virus with short-stalk NA, Vac2/L4NA, did not elute erythrocytes within 8 h (Fig. 3A). Similarly, the NA-stalk deglycosylated virus, Vac2/P0NA∆G, also failed to elute chicken erythrocytes, suggesting that the loss of *N*-glycosylation in the NA-stalk domain, rather than stalk truncation, reduces the erythrocyte elution activity of the virus. Since Arai et al. (35) reported that the deglycosylation or truncation in the NA stalk domain has a minor effect on the substrate-binding affinity of the catalytic site, the NA activity was also evaluated using a small compound, MUNANA. The data were fitted to the Michaelis-Menten equation via non-linear regression, and the Michaelis constant (*K*_m_) was calculated for each of the viruses. The *K*_m_ values were 73.7 ± 5.38 µM for Vac2/P0NA, 83.8 ± 4.79 µM for Vac2/P0NA∆G, and 68.3 ± 5.08 µM for Vac2/L4NA, indicating that their comparable affinities with the small substrate (Fig. 3B). Although the maximum velocity of the reaction (*V*_max_) for Vac2/L4NA was higher than the others, this rather reflects the higher input of viruses in the assay. These results demonstrate that although the deglycosylation and truncation in the NA stalk domain do not affect its sialidase activity to small substrates, they remarkably affect the erythrocyte elution activity, suggesting the reduction of activity to bulky substrates.

Next, the effect of the Y65H mutation on elution activity using a pair of Y65H mutants (Vac2/P0NA-65H and Vac2/P0NA∆G-65H; Fig. 3C) was assessed. The erythrocyte elution activity of Vac2/P0NA-65H was relatively lower than that of Vac2/P0NA, whereas the phenotypic change induced by glycosylation deficiency was evident in the elution activity of Vac2/P0NA∆G-65H. Thus, the impact of the Y65H mutation in the NA-stalk domain was limited to the erythrocyte elution activity of the viruses.

### Potential *N*-glycosylation sites on the NA stalk of rgVac2-P0NA were occupied

Because the loss of potential *N*-glycosylation sites in the NA-stalk domain increased decreased erythrocyte elution activity, two hypotheses arose: decreasing NA intake in a virion or functional changes of NA via deglycosylation at the NA stalk. First, the amount of NA protein present in the virus particles was considered. To detect NA proteins using a commercial antibody, plasmids coding FLAG (DYKDDDDK)-tagged NA proteins were generated as described by (36; Fig. S3A). A set of plasmids coding FLAG-tagged NA (Vac2/P0NA-FLAG, Vac2/P0NAΔG-FLAG, and Vac2/L4NA-FLAG) were used for virus rescue with the Vac-2 backbone to obtain higher titers of these viruses. The relative NA/NP ratios of the glycosylation-deficient virus and NA-stalk truncated virus were compared with the virus harboring full-stalk NA using dot blotting. The relative NA intake in the virions was calculated as 1.48 for Vac2/P0NAΔG-FLAG and 0.74 for Vac2/L4NA-FLAG; however, these values did not correlate with the ability of potential glycosylation sites on the NA-stalk domain (Fig. S3B).

Second, to validate the glycan occupancy of the NA-stalk domain, the molecular weights of NA proteins were compared via western blotting (Fig. 3D). The molecular weight of Vac2/P0NA-FLAG was larger than that of Vac2/P0NAΔG-FLAG under nontreated conditions. However, after treatment with peptide *N*-glycosidase F (PNGase F), which removes all *N*-glycans from the NA proteins, the electrophoretic mobility of Vac2/P0NA-FLAG and Vac2/P0NAΔG-FLAG was comparable. It should be noted that the faint band was obtained at approximately 65 kDa in Vac2/P0NAΔG-FLAG without PNGase F treatment because this NA was prone to be aggregated due to the deglycosylation, which was also supported by the presence of another band at the top of the resolving gel. These results indicate that P0NA-FLAG has a higher number of *N*-glycosylations compared with P0NAΔG-FLAG, suggesting that the stalk domain of P0NA-FLAG is glycosylated with at least one *N*-glycan.

**Fig 4:**
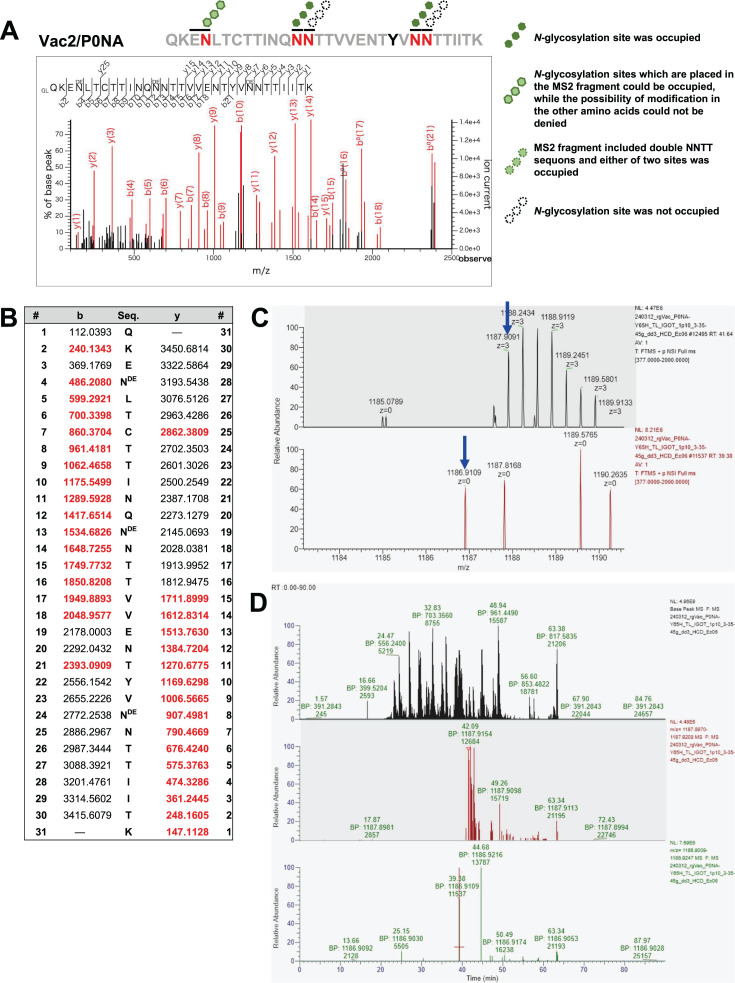
MS2 spectra of the peptide of P0NA with three glycans and mass chromatograms of precursor ions to identify site occupancy (**A**) MS2 spectra of the peptide of P0NA containing three glycans. (**B**) Molecular mass of b and y ion fragments derived from the peptide of P0NA. Red letters indicate b and y ion fragments detected in this analysis, whereas black letters refer to the calculated molecular weights based on the amino acid sequence. (**C**) The monoisotopic peak of MS1 corresponding to the less-glycosylated peptide was not observed. The blue arrow indicates the estimated molecular weight of the less-glycosylated peptide. (**D**) Mass chromatogram of the precursor ion corresponding to the less-glycosylated peptide was not detected.

**Fig 5:**
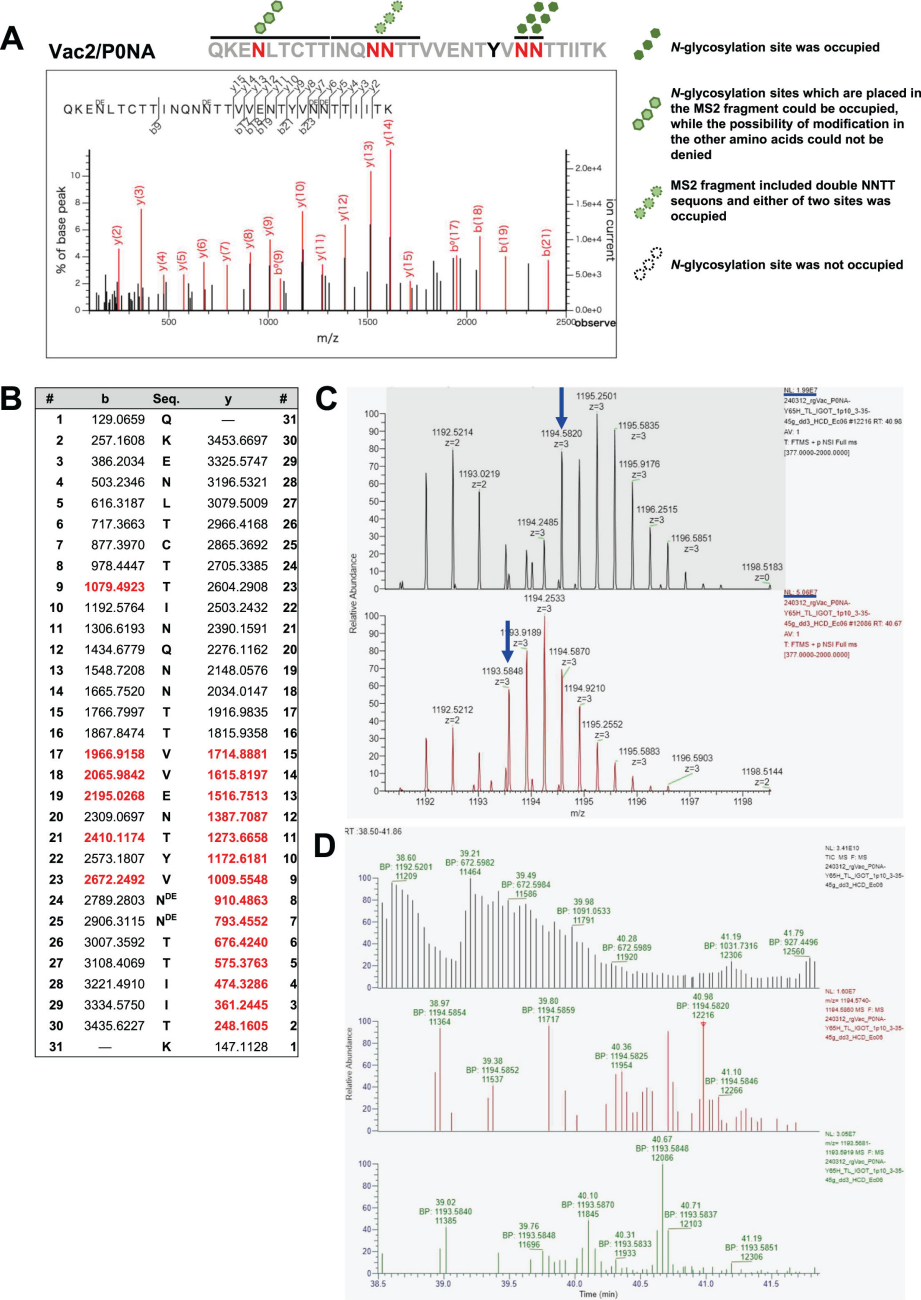
MS2 spectrum of the peptide of P0NA with four glycans and mass chromatograms of precursor ions to identify site occupancy. (**A**) MS2 spectrum of the peptide of P0NA containing four glycans. (**B**) Molecular mass of b and y ion fragments derived from the peptide of P0NA with four glycans. Red letters indicate b and y ion fragments detected in this analysis, whereas black letters refer to the calculated molecular weights based on the amino acid sequence. (**C**) The monoisotopic peak of MS1 corresponding to the less-glycosylated peptide, which had a smaller molecular mass by 2.99 Da (*m*/*z* 0.997 [*z* = +3]; two blue arrows), was observed. (**D**) Mass chromatogram of precursor ions shows that the less-glycosylated peptide with three glycans (40.67 min; green) had a shorter LC retention time compared with the peptide with four glycans (40.98 min; red).

To comprehensively investigate site-specific glycan occupancy on the NA-stalk domain, IGOT-LC/MS analysis was performed (37, 38). Among the tryptic digests identified, all five potential *N*-glycosylation sites were included in a single peptide (QKENLTCTTINQNNTTVVENTYVNNTTIITK). In the IGOT analysis, glycan-liberated asparagines were converted into ^18^O-containing aspartic acids by digestion with PNGase F in H_2_^18^O. Therefore, glycosylated peptides could be differentiated from nonglycosylated peptides by a molecular mass shift of 2.988261 Da per glycosylation site. In the peptides derived from P0NA, three or four sites were generally occupied among five N-!P-(S|T) (!P = any amino acid except proline) sites, and peptides with fewer than three *N*-glycans were not identified. In MS1 spectra, a total of six distinct peptides covering the five potential *N*-glycosylation sites of P0NA were identified via Mascot searching (Fig. 4 and 5; Fig. S4 to S7). In a peptide glycosylated with three asparagine residues, the *N*-glycosylations on N47, N56, and N67 were confirmed by the b and y ions in MS2 spectra (Fig. 4A and B). Subsequently, a monoisotopic peak for non- or less-glycosylated peptides, which have a smaller molecular mass than the glycosylated peptide by 2.988261 Da, was searched for in MS1 spectra. However, no specific ions corresponding to less-glycosylated peptides were obtained in the MS1 spectra, indicating that peptides with two glycans were not identified (Fig. 4C and D). Moreover, two peptides with four isotope-coded glycosylation site-specific tagging (IGOT) labels were identified through database searching using Mascot, and double glycosylation at either of the NNTT sequons was suspected in these peptides. In one of the two peptides, consecutive glycosylations at N67 and N68 were identified based on the y ion fragments in the MS2 spectrum, whereas N47 and either N56 or N57 were occupied in the peptide (Fig. 5A and B). In the other peptide, the b and y ion fragments were not observed at the NNTT sequon; therefore, the site-specific glycan occupancy of N56 and N57 was not confirmed (Fig. S7). To estimate the abundance ratio of peptides with three versus four *N*-glycans, the mass chromatograms of the precursor ions were analyzed. A monoisotopic peak with *m*/*z* 1194.5820 (*z* = +3) was obtained, which was assigned to the peptide with four glycans, whereas a larger peak with *m*/*z* 1193.5848 (*z* = +3), corresponding to the peptide with three glycans, was also detected (Fig. 5C). The latter signal exhibited a shorter LC retention time, confirming it as the peak of the glycosylated peptide with three glycans (Fig. 5D). The ratio of peak intensities of monoisotopic ions in the MS1 spectra indicated that peptides with four *N*-glycans (1.99 E7; Fig. 5C) constituted 40% of those with three glycans (5.06 E7; Fig. 5C). These results demonstrate that the NA-stalk domain of P0NA is occupied by three or four *N*-glycans. Additionally, at least one of the asparagine residues in the NNTT sequons, observed in N56/57 and N67/68, was glycosylated.

The site-specific glycan occupancy was also assessed for P0NA-Y65H to confirm the effect of the Y65H mutation on glycosylation in the NA-stalk. As observed with P0NA, three or four of the five potential *N*-glycosylation sites were occupied, and peptides with fewer than three *N*-glycans were not detected (Fig. S8 to S12). Moreover, the ratio of peak intensities of precursor ions corresponding to triple and quadruple glycosylated peptides indicated that peptides with four glycans reached a level comparable with those with three glycans (Fig. S8 to S11).

These results indicate that the potential *N*-glycosylation sites on the NA-stalk domain of P0NA were occupied. Furthermore, the Y65H mutation did not affect the overall glycosylation of the NA-stalk domain.

### Various contributions of *N*-glycosylations to NA function among the sites

**Fig 6:**
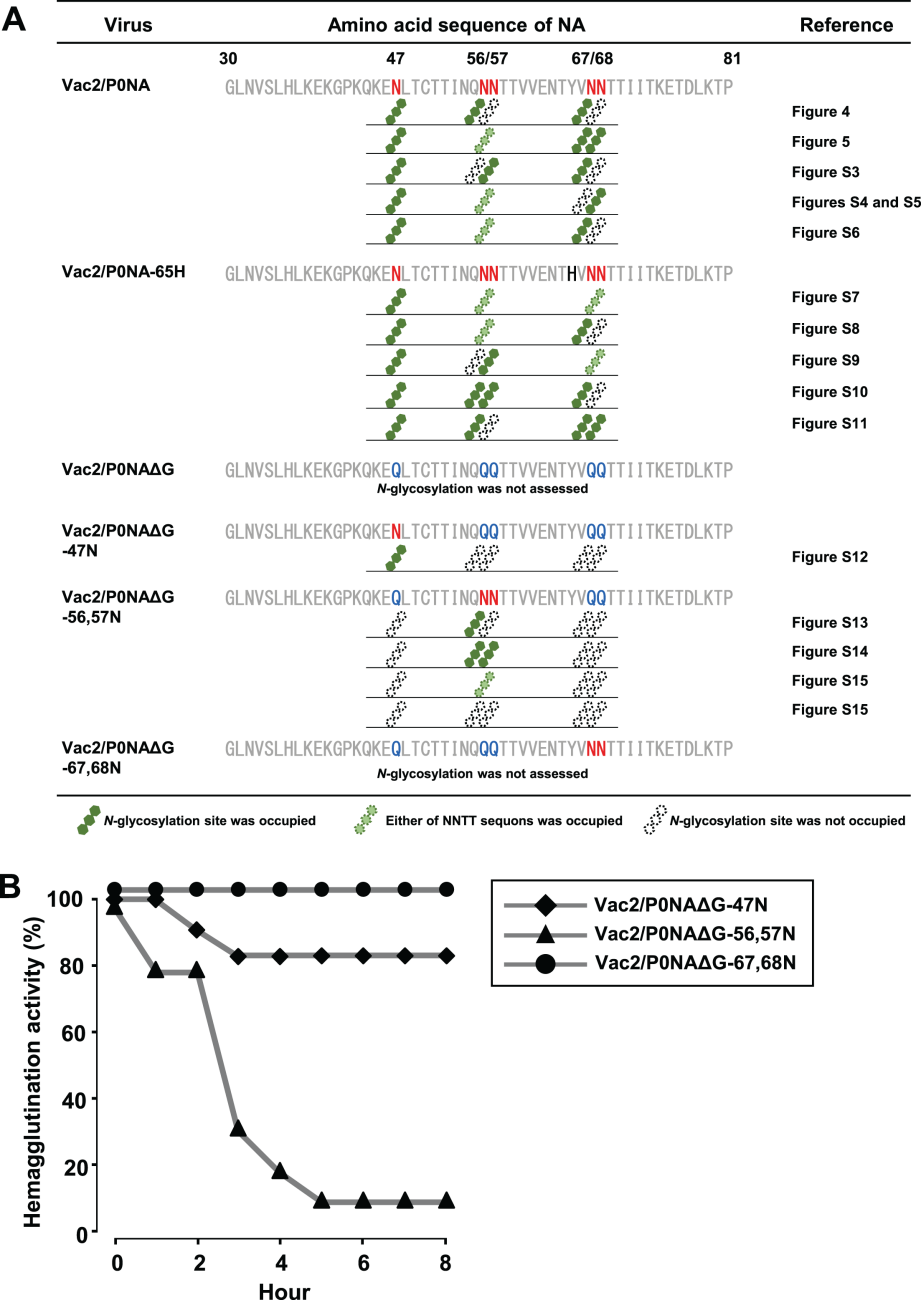
Glycosylation patterns of potential *N*-glycosylation sites in the NA-stalk domain of Vac2 mutants. (**A**) Amino acid sequences of the NA-stalk domain for the viruses constructed in this study are listed. Glycosylation patterns were summarized based on LC-MS/MS analysis. (**B**) Erythrocyte elution activity of Vac2 mutants with the Y65H substitution in the NA was assessed.

Because three or four of the five potential *N*-glycosylation sites in the stalk domain of P0NA were occupied with *N*-glycans, the functional contribution of each *N*-glycosylation site (amino acids 47, 56, 57, 67, and 68) to erythrocyte elution activity was assessed. To this end, mutant viruses with partially restored *N*-glycosylation sequons through Q/N substitution in P0NAΔG were rescued (Fig. 6A). The virus harboring potential glycosylation sites at N56 and N57, Vac2/P0NAΔG-56,57N, showed comparable erythrocyte elution activity with the virus with full-stalk NA, rgVac2/P0NA (Fig. 6B). Conversely, the virus harboring potential glycosylation sites at N67 and N68, Vac2/P0NAΔG-67,68N, did not elute erythrocytes, and the virus with one glycosylation site at N47, Vac2/P0NAΔG-47N, showed intermediate elution activity over 8 h (Fig. 6B). These results indicate that the potential *N*-glycosylation sites at N56 and N57 significantly contribute to the high erythrocyte elution activity of the virus, whereas the site at N47 less contributed to this activity. In addition, the results implied that the presence or absence of glycans at N67 and N68 does not affect the erythrocyte elution activity. Accordingly, it was speculated that the glycan occupancy at N47 and N56/57 of the mutants affects erythrocyte elution activity, and therefore, the site-specific glycan occupancy of two mutants, Vac2/P0NAΔG-47N and Vac2/P0NAΔG-56,57N, was assessed via LC-MS/MS. The b and y ion fragments corresponding to *N*-glycosylation at N47 of the NA-stalk derived from Vac2/P0NAΔG-47N were not identified via MS/MS. However, the precursor ion obtained in the MS1 spectrum indicated an ^18^O label in a mono-asparagine residue in the peptide, suggesting that the potential *N*-glycosylation site at N47 of P0NAΔG-47N was occupied (Fig. S13). Additionally, the nonglycosylated peptide was not identified in the mass chromatogram, indicating that glycan occupancy at N47 of P0NAΔG-47N reached 100%. Moreover, peptides with one or two *N*-glycans in the NA-stalk domain of P0NAΔG-56,57N were detected via MS/MS (Fig. S14 to S16). Among these peptides, glycosylation at N56 was identified, but there was no evidence of single glycosylation at N57. Precursor ions corresponding to nonglycosylated peptides were detected, with their peak intensity being 20% of that of the single-glycosylated peptides. Furthermore, the intensity of ion peaks corresponding to double-glycosylated peptides was 40% lower than that of single-glycosylated peptides (Fig. S14 to S16). These results indicate that the potential *N*-glycosylation sites at N47 of P0NAΔG-47N, and N56 and/or N57 of P0NAΔG-56,57N were generally occupied. Thus, the functional difference in erythrocyte elution activity was not due to glycan occupancy alone. Rather, *N*-glycosylation at each site contributed differently to erythrocyte elution activity: glycosylation deficiency at N56 and N57 greatly reduced elution activity, whereas deficiency at N47 had a milder effect, and deficiency at N67 and N68 did not reduce erythrocyte elution activity.

### Comparison of NA-stalk domain of N7 NA AIVs

**Fig 7:**
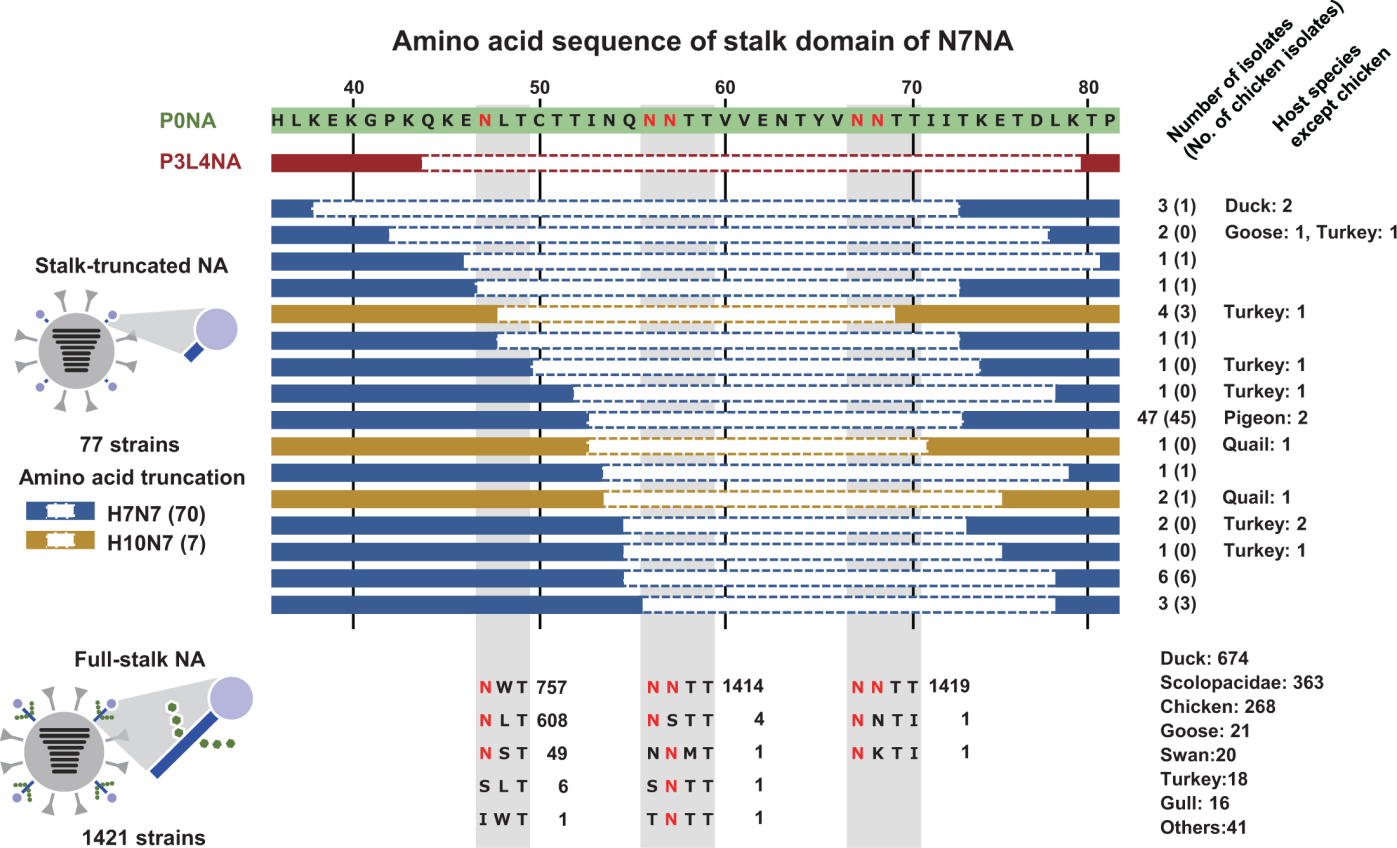
Overview of multiple potential *N*-glycosylation sites in the NA-stalk domain of N7NA proteins and patterns of amino acid truncation in natural isolates amino acid sequences of N7NA proteins covering the stalk domain were obtained from the Global Initiative on Sharing All Influenza Data (GISAID, https://gisaid.org/) and aligned with Vac2-P0NA and Vac2-P3L4NA. The sequences of stalk-truncated NAs are indicated, and the amino acid substitutions at the *N*-glycosylation sites in full-stalk NAs are summarized.

Finally, the potential *N*-glycosylation motifs in the NA-stalk domain were compared among field-isolated N7 NA AIVs. A previous study has shown that NA-stalk deletion in AIVs reduces erythrocyte elution activity, which correlates with increased viral pathogenicity in chickens (22). In the present study, *N*-glycosylations at N56 and N57 were found to contribute most significantly to erythrocyte elution activity among the five potential sites. Therefore, it was hypothesized that the loss of *N*-glycosylations at these positions might have a greater impact on the adaptation of N7 NA to chickens. In the consensus amino acid sequences of the NA-stalk domain, the five potential *N*-glycosylation sites confirmed in P0NA were included in the 34-amino-acid truncation of P3L4NA (Fig. 7). Of these 5 potential glycosylation sites, 4 (at N56/57 and N67/68) were consistently included in the deletion sites of the stalk-truncated NA, despite the truncation position or length varying among 16 patterns. Conversely, some strains lost potential glycosylation sites at N47 due to amino acid substitutions. However, at least one potential site in either of the two adjacent glycosylation sites (NNTT sequons) observed in N56/57 and N67/68 was conserved among the full-stalk NA. These results suggest that amino acid truncation, which includes potential *N*-glycosylation sites at N56/57 and N67/68 in the NA-stalk, is beneficial for viral adaptation to Galliformes in natural settings. Together with the functional analysis using Vac2 strains, these NA-stalk truncations of N7 NA result in deglycosylation of the stalk and increased viral pathogenicity in chickens.

## DISCUSSION

Amino acid truncation in the stalk domain of the NA protein has been identified as a molecular marker for AIVs that acquire a highly pathogenic phenotype in chickens, in addition to the polybasic amino acid sequences at the HA cleavage sites (21–23, 30). Viruses with stalk-truncated NA have indeed been isolated as a result of virus passages in quails and chickens (14–17). Previous studies of a genetically modified H7N7 nonpathogenic AIV (rgVac2sub-P0) in chickens also yielded an HPAIV with a 34-amino-acid deletion in the NA-stalk domain, which included five potential *N*-glycosylation sites (Vac2sub-P3L4; (30)). However, the specific mechanisms by which NA-stalk truncation or deglycosylation contribute to increased viral pathogenicity remain unclear. In the present study, the functional significance of NA-stalk deglycosylation was investigated on viral pathogenicity in chickens. The findings show that the loss of glycosylation in the NA-stalk of the H7N7 AIV leads to increased pathogenicity and decreased elution activity against chicken erythrocytes, similar to what is observed with NA-stalk truncation. These results suggest a novel hypothesis: the loss of glycosylation in the NA-stalk domain, rather than simply a reduction in the number of amino acids, significantly contributes to enhanced pathogenicity in chickens.

For certain decades, the “limited access theory” has been used to explain the reduced sialidase activity of short-stalk NA proteins. According to this theory, the shorter height of the NA stalk compared with HA restricts access to cellular sialylated glycans, impeding the enzymatic ability to interact with “bulky” substrates (24). This theory aligns with observations that viruses with stalk-truncated NA exhibit low erythrocyte elution activity (30, 34). However, recent research suggests that the limited access theory may not fully account for the reduced NA activity of short-stalk NA proteins (25). The present study found that the loss of glycosylation in the NA-stalk domain, rather than the shortened height of the NA stalk, led to decreased elution activity against bulky substrates like chicken erythrocytes without alteration of NA enzymatic activity to a small compound (Fig. 3A and B). The observation agreed with the results of MUNANA assays with NA stalk-truncated or deglycosylated strains in several studies (35, 39, 40). Perhaps, the affinity of NA head domain to bulky substrates was affected by the NA-stalk modification. Generally, protein glycosylation can decrease root-mean-square fluctuations, enhance global protein mobility, or increase protein dynamics and flexibility (31, 41–43). A molecular dynamics simulation of an NA protein has demonstrated that glycans on the protein, in general, decrease the protein rigidity. However, the effects of glycosylation on the protein stability may not be consistent throughout the protein and may differentially affect the two functional domains; for example, the glycosylation of NA stabilizes the NA head domain while minimally affecting the stalk domain (44). Likewise, deglycosylation at the stalk domain can reduce the stability of NA head domain. Thus, the loss of glycosylation in the NA-stalk plays a critical role in increasing the fluctuation of the NA head and reducing the enzymatic activity to balky substrates.

Deglycosylation or truncation encompassing potential *N*-glycosylation sites at the NA stalk mainly contributes to decreasing erythrocyte elution activity of the virus (35, 45, 46), whereas the functional contribution of *N*-glycosylations on the NA stalk was still controversial due to the less information of the site-specific glycosylation. The present study highlighted the site-specific contributions of *N*-glycosylations on the NA-stalk to the erythrocyte elution activity of viruses. Glycosylation at N56 and N57 was associated with higher elution activity, whereas glycosylation at N68 and N69, which are closer to the head domain, did not affect elution activity. Additionally, glycosylation at N47, located near the transmembrane domain, contributed intermediate effects to erythrocyte elution activity. These observations suggest that the position of *N*-glycosylation sites influences the stability of the NA head, thereby affecting its activity on bulky substrates. Interestingly, field isolates of N7 NA proteins often exhibit truncation of the stalk domain, including the *N*-glycosylation sites at N56/57 and N67/68. This field observation supports the functional significance of *N*-glycosylations in the NA-stalk and may be related to the varying contributions of these glycans to NA function across different sites. Overall, these results underscore the importance of structural and functional analyses of NA glycans to understand how NA-stalk deletion affects viral sialidase activity and its implications for the emergence of HPAIVs.

A few recent studies have revealed the site-specific occupancy of glycans in recombinant NA or purified viruses using mass spectrometry (47, 48). She et al. (47) reported that all potential *N*-glycosylation sites located in the head domain of N1 NA were occupied, whereas glycans were identified on only some of the *N*-glycosylation sites in the stalk domain. Thus, this study provides a comprehensive view of *N*-glycan occupancy on the NA stalk. *N*-glycans are attached to asparagine residues within N-!P-(S|T) motifs (!P = any amino acid except proline). However, the mere presence of such motifs is not sufficient for *N*-glycosylation; the accessibility of glycan transferases and the structural topology of proteins during synthesis also play crucial roles. It is known that transmembrane domains can influence the glycosylation of these sequons, particularly when sequons are located fewer than 12–14 residues from a transmembrane region (49). The high glycan occupancy at N47 suggests that 17 residues from the transmembrane region did not hinder the accessibility of oligosaccharyltransferase (OST) in the NA stalk, which is consistent with previous reports. Interestingly, double glycosylation on overlapping sequons, NNTT, was observed at N56/57 in P0NA, P0NA-65H, and P0NAΔG-56,57N, as well as at N67/68 in P0NA and P0NA-65H (Fig. 6A). Overlapping sequons like NNTT are rarely observed in host endogenous proteins and, when present, are typically glycosylated at only one asparagine residue. For example, the NNST sequence in human cholinesterase is glycosylated only at the second asparagine residue, and the NNTS sequence in yeast invertase is glycosylated only at the first asparagine (50, 51). These observations can be explained by steric hindrance caused by bulky substrates attached to one of the asparagine residues, which prevents OST from accessing the second site. Conversely, overlapping sequons are sometimes observed in viral glycoproteins, including the HA and NA of influenza A viruses (47, 48). For example, the overlapping sequon NNTT in the GP3 protein of equine arteritis virus was glycosylated at both asparagine residues, despite being located just two amino acids downstream of an *N*-terminal signal peptide, including a transmembrane region (52). This observation suggests that the signal peptide of GP3 or a shorter distance (five amino acids) between the transmembrane region and the overlapping sequon may facilitate double glycosylation. In the present study, however, the NNTT sequons at N67/68 of the NA-stalk domain were glycosylated at both sites, although this sequon is 37 amino acids from the transmembrane domain. This indicates that a different mechanism in NA proteins, compared with GP3 proteins, might facilitate the double glycosylation of overlapping sequons. Factors such as the flexibility of the NA-stalk domain or specific amino acid sequences may play a role. Additionally, because the virus specimens were prepared using chicken embryonic eggs, the preference of chicken OST for certain acceptor sequons might also influence the double glycosylation observed on the NNTT sequons.

Amino acid deletions in the NA-stalk domain, including some potential *N*-glycosylation sites, have been observed in several isolates of N1–3 and N5–7 subtypes from terrestrial birds such as chickens and turkeys (19, 20). In N7 NA, all patterns of amino acid deletions in the NA-stalk domain of natural isolates included more than one potential *N*-glycosylation site, although the lengths of the deletion sites varied among strains (Fig. 7). Conversely, only a few strains lost a potential *N*-glycosylation site due to amino acid substitutions in the stalk. These observations suggest that the loss of glycosylation in the NA-stalk domain in natural settings is primarily achieved through amino acid deletions rather than substitutions. Kawasaki et al. (53) found that the frequency of insertions/deletions was 10 times lower than that of nonsynonymous mutations in the coding regions of NA genes over 15 passages *in vitro* of an influenza A virus. The finding supports the idea that a single amino acid deletion in the NA-stalk domain, rather than multiple nonsynonymous mutations, is more likely to result in the loss of glycosylation in field isolates. Once a nucleotide deletion in the NA-stalk coding region occurs and a NA-stalk truncated virus emerges during replication in chicken respiratory tracts, the NA-stalk truncated virus is likely to become dominant due to its advantage in viral proliferation within the chicken host. In other words, the presence of multiple *N*-glycosylations in the NA-stalk domain may provide functional redundancy that benefits virus evolution in natural hosts.

In the present study, several NA-stalk variants were rescued from chickens inoculated with L4/P0NAΔG without requiring multiple passages (Tables S3 and S4). By introducing a set of nucleotide substitutions from AAU/AAC (asparagine) to CAG (glutamine) in the stalk-coding domain to create a glycosylation-deficient mutant of P0NA, a stem-loop structure was predicted at the substitution site in the viral RNA of P0NAΔGlyco. Secondary RNA structures are known to cause slippage or dissociation of the viral polymerase (54), and a stem-loop structure at HA cleavage site of H5 and H7 subtypes is essential for the poly-adenine insertion, which leads to serial basic amino acid residues at these sites (55). Similarly, the RNA secondary structure in the stalk-coding region of NA may enhance the propensity for polymerase slippage and increase the likelihood of nucleotide truncation. Based on the predicted RNA secondary structures, the Y65H mutation (a change from uracil to cytosine) located just upstream of the glycosylation-deficient site, disrupted the stem-loop structure in the NA-stalk coding region of P0NAΔGlyco. As Y65H mutations in N7 NA have not been reported in field isolates according to the GISAID public database, this mutation likely results from the removal of the instability associated with genetically modified NA genes. Consequently, the abolition of the RNA secondary structure is thought to contribute to the higher genetic stability of L4/P0NAΔG-65H during viral replication in chickens and embryos.

The functional balance between glycan binding by HA and cleaving by NA is also considered in the host specificity of AIVs (19, 45, 46). During the passage to obtain the L4 mutant, amino acid substitutions in HA were also observed, in addition to the stalk truncation in NA (30). It should be noted that no further mutation in HA genes was obtained from the recovered viruses in the experimental inoculation with the L4 mutant in the present study. The mutations E227G and I388T in HA observed in the previous study were essential for the intravenous pathogenicity in chickens and E227G especially contributed to the reduced receptor binding avidity (30). The reduction of binding to the receptor by the mutation in HA was perhaps later balanced with the reduced function of NA by deglycosylation or truncation in the stalk domain, leading to the efficient replication of L4 and L4/P0NAΔG in chickens. In theory, lower NA activity compared with the receptor binding avidity by HA reduces the moving of the virions on the sialic acid-coated surface *in vitro* (56). Since the repertoire of sialic acid-containing glycans varies between species, further studies are needed to address how the reduced function of NA benefits viral replication in chickens.

In conclusion, the present study introduces a novel theory that the loss of glycosylation in the stalk domain of NA, rather than amino acid truncation, enhances viral pathogenicity in chickens. This theory is supported by a combination of site-specific *N*-glycosylation analysis and virological assays. Chickens inoculated with a virus harboring a glycosylation-deficient NA-stalk experienced a more rapid clinical course compared with those inoculated with a virus harboring a glycosylation-retained NA-stalk. The erythrocyte elution assay demonstrated that the loss of glycosylation in the NA-stalk domain, rather than amino acid truncation, contributed to decreased elution activity of the viruses. This suggests that glycosylation deficiency in the NA-stalk domain results in increased pathogenicity in chickens. In natural settings, amino acid truncation in the NA-stalk occurs more frequently than multiple amino acid substitutions at potential glycosylation sites. Consequently, viruses with glycosylation-deficient NA-stalks and truncated amino acids in the stalk are likely selected due to their enhanced replication ability in chickens. However, the specific mechanisms by which decreased NA activity enhances viral pathogenicity in chickens remain unknown. Further studies are needed to elucidate how glycan-deficient NA stalks enhance virus attachment or replication in chickens.

## MATERIALS AND METHODS

### Viruses and cells

A low pathogenicity, AIV, A/duck/Hokkaido/Vac-2/2004 (H7N7) (Vac2; genetic information accession numbers: AB243417–AB243424; (57)) was used. The gene constellations and nucleotide sequences of all eight segments are listed in Table S12. These viruses were propagated in 10-day-old embryonated chicken eggs at 35°C for 48 h, and the infectious allantoic fluids were used as virus stocks.

Madin-Darby canine kidney (MDCK) cells were maintained in minimum essential medium (MEM; Shimadzu Diagnostics Corporation, Tokyo, Japan) supplemented with 0.3 mg/mL L-glutamine (Nacalai Tesque Inc., Kyoto, Japan), 100 U/mL penicillin G (Meiji Seika Pharma Co., Ltd., Tokyo, Japan), 0.1 mg/mL streptomycin (Meiji Seika Pharma), 8 µg/mL gentamicin (Takata Pharmaceutical Co., Ltd., Saitama, Japan), and 10% fetal bovine serum (Merck KGaA, Darmstadt, Germany). Human embryonic kidney (HEK) 293T cells were maintained in pyruvate-free Dulbecco’s modified Eagle’s medium (Thermo Fisher Scientific, Inc., Waltham, MA, USA) supplemented with the above-mentioned antibiotics and 10% fetal bovine serum (Nichirei Biosciences Inc., Tokyo, Japan). Both cell types were maintained at 37°C in a 5% CO_2_ atmosphere.

### Virus titration

For virus titration, 10-fold dilutions of the virus stocks and organ homogenates were inoculated into confluent monolayers of MDCK cells and incubated at 35°C for 1 h. The virus solution, including unbound viruses, was removed from the cells, and the cells were washed with sterilized phosphate-buffered saline (PBS). The cells were then cultured in serum-free MEM containing 1.0 µg/mL of acetylated trypsin (Merck) at 35°C for 72 h. The infectious titers of the viruses were expressed as 50% tissue culture infectious dose (TCID_50_), determined using the method reported by Reed and Muench (58), by observing the cytopathic effect (CPE) in virus-infected cells.

### Pathogenicity assessment of viruses in chickens

Fertilized eggs of conventional chickens (*Gallus gallus domesticus*) were obtained from Iwamura Poultry (Niigata, Japan) and hatched. Six-week-old chickens were used for the pathogenicity assessment of the viruses. Serum samples were collected from the chickens before the challenge to confirm the absence of specific antibodies against the challenge virus using standard hemagglutination-inhibition (HI) test protocols with 25 µL of serum. All chickens were intranasally inoculated with 10^4.0^ TCID_50_ of viruses. To observe survival rates and clinical manifestations, six chickens were challenged with each virus and monitored for 14 days. Once a chicken exhibited severe neurological symptoms, it was euthanized with an overdose of thiopental sodium (Ravonal, 150 mg/kg; Nipro ES Pharma Co., Ltd., Osaka, Japan) administered intravenously, based on a humane endpoint. The number of euthanized chickens was recorded the following day and included in the survival curve. Lung and brain samples were collected from both deceased and euthanized chickens for genetic analysis of the recovered viruses. Additionally, a serum sample was collected from surviving chickens during the experimental period, and HI tests were performed using Vac2 to confirm seroconversion. For virus titration, blood samples were collected from chickens at 3 dpi, and the chickens were subsequently euthanized. Tissue samples from the brain, trachea, lung, liver, kidney, and colon were collected and homogenized using a Multi-Beads Shocker (MB3000, Yasui Kikai Corp., Osaka, Japan) to prepare 10% suspensions in MEM. Infectivity titers for blood and tissue samples were measured using MDCK cells as described above. All infected animals were housed in self-contained isolator units (Tokiwa Kagaku Kikai Co., Ltd., Tokyo, Japan) within an ABSL3 biosafety facility at the Faculty of Veterinary Medicine, Hokkaido University, Hokkaido, Japan.

### Reverse genetics

The NA genes of Vac2 mutants were amplified and cloned into the pHW2000 vector (59) using the In-Fusion HD Cloning Kit (Takara Bio Inc., Shiga, Japan), following the manufacturer’s protocol. To assess the molecular weight of NA proteins, a nucleotide sequence encoding a FLAG (DYKDDDDK) tag with a single glycine linker (G) was inserted downstream of the C-terminus coding region of NA. This insertion was followed by 157 nucleotides from the 3ʹ end of the ORF, which includes a noncoding region consisting of a total of 183 nucleotides of the packaging signal (36, 60). Individual nucleotide substitutions in the NA gene segments were introduced via site-directed mutagenesis using the QuikChange II Site-Directed Mutagenesis Kit (Agilent Technologies, Inc., Santa Clara, CA, USA) or KOD FX Neo DNA Polymerase (Toyobo, Osaka, Japan), according to the manufacturer’s instructions. The other seven gene segments from Vac2 and Vac2-P3L4 were previously cloned into pHW2000 (27, 30). The viruses listed in Table S1 were rescued via reverse genetics as described previously (59). All the nucleotide sequences of generated viruses are listed in Table S2.

### Sanger sequencing of NA genes

Virus RNA was extracted from the allantoic fluid of virus-infected chicken embryos or organ homogenates using TRIzol LS Reagent (Thermo Fisher Scientific), according to the manufacturer’s protocol. The extracted RNA was reverse-transcribed with M-MLV Reverse Transcriptase (Promega Corp., Madison, WI, USA) or SuperScript III Reverse Transcriptase (Thermo Fisher Scientific) using the Uni 12 primer (61). The NA segments were then amplified with KOD FX Neo DNA Polymerase (Toyobo) and gene-specific primers (61). Nucleotide sequences of the PCR products and plasmids were determined using the BigDye Terminator v3.1 Cycle Sequencing Kit (Thermo Fisher Scientific) and the Applied Biosystems 3500 Genetic Analyzer (Thermo Fisher Scientific). The sequences were analyzed using GENETYX ver. 15 (Nihon Server Corp., Tokyo, Japan).

### Whole genome sequence

Whole genomes of viruses recovered from L4/P0NAΔG-inoculated chickens (#31, 33–36) were analyzed. For next-generation sequencing (NGS) of the viruses, the primer sets described by Ip et al. (62) were used to amplify the whole genome of AIV for NGS analysis together with three primer sets that specifically improve the yield of PB2, PB1, and PA segments described by Hew et al. (63). Oxford Nanopore libraries were prepared using the NEB Ultra II End Repair/dA-Tailing Module (New England Biolabs, Ipswich, MA, USA) and sequenced on Flongle using the Nanopore Direct cDNA sequencing kit or Ligation Sequencing kit V14 (Oxford Nanopore Technologies, Oxford, England). The obtained reads were mapped and assembled using FluGAS version 2 (World Fusion, Tokyo, Japan). The frequency of minor variants was analyzed based on the sequence data with a minimum coverage of 3,500 reads for the NA segment and 500 reads for the other segments.

### Immunoprecipitation

One milliliter of infectious allantoic fluid containing viruses was mixed with 200 ng of biotinylated anti-H7HA monoclonal antibodies (224/4; previously established in our laboratory using A/duck/Hokkaido/Vac-2/2004 [H7N7]; [64]). After incubation at 37°C for 1 h, the complexes were captured with Dynabeads MyOne Streptavidin T1 (Thermo Fisher Scientific) at 4°C for 1 h. After washing, virus antigens were eluted from the magnetic beads and disrupted using 100 mM citric acid containing 1% Triton X-100. The supernatant was mixed with an equal volume of ice-cold acetone and incubated at −20°C for 1 h to precipitate proteins. After centrifugation at 13,000 × *g* at 4°C for 15 min, the pellets were dried at room temperature for 30 min. The pellets were then incubated with 20 µL of denaturation buffer (1 M Tris-HCl [pH 8.6], 1% SDS, 1.5% 2-mercaptoethanol) at 4°C overnight to dissolve them.

### Glycosidase digestion

Immunoprecipitated samples were treated with PNGase F to release the *N*-glycans from the NA proteins. The samples were mixed with an equivalent amount of 5% NP-40 for stabilization and incubated at 37°C for 20 min with PNGase F PRIME (N-Zyme Scientifics, Doylestown, PA, USA).

### SDS-PAGE and western blotting

The samples were mixed with an equivalent amount of 2× Laemmli sample buffer (125 mM Tris-HCl [pH 6.8], 4% SDS, 20% glycerol, 0.02% bromophenol blue, 10% 2-mercaptoethanol). SDS-PAGE was performed using a 10% TGX FastCast Acrylamide gel (Bio-Rad Laboratories Inc., Hercules, CA, USA) at a constant voltage of 200 V for 35 min under reducing conditions. The resolved proteins were transferred to an Immobilon-P PVDF membrane (Merck). The membrane was blocked with PVDF Blocking Reagent for Can Get Signal (Toyobo). Subsequently, the membrane was probed with an anti-DDDDK-tag monoclonal antibody (FLA-1, 1:1000 dilution; Medical & Biological Laboratories Co., Ltd., Tokyo, Japan) and a goat anti-mouse IgG antibody conjugated with horseradish peroxidase (1:5000 dilution; Bio-Rad). Proteins were detected using the chemiluminescence substrate ImmunoStar LD (Fujifilm Wako Pure Chemical Corp., Osaka, Japan) and visualized with a LuminoGraph I (WSE-6100, Atto Corp., Tokyo, Japan).

### Dot blotting

For dot blotting, an Immobilon-P PVDF membrane (Merck) was immersed in methanol and ultrapure water to wet it. Then, 1 µL of immunoprecipitated virus samples was spotted onto the membrane and allowed to dry. The membrane was blocked with PVDF Blocking Reagent for Can Get Signal (Toyobo). Subsequently, the membrane was probed with either an anti-DDDDK-tag monoclonal antibody (FLA-1, 1:1000 dilution; Medical & Biological Laboratories) or an anti-NP monoclonal antibody (183/5, 1:1000 dilution; previously established in our laboratory using A/swine/Hong Kong/10/1998 [H9N2]) as primary antibodies. After incubation with a goat anti-mouse IgG antibody conjugated with horseradish peroxidase (1:5000 dilution; Bio-Rad), proteins were detected using the chemiluminescence method described above. The chemiluminescent density was quantified as previously described by Ohgane and Yoshioka (65).

### Site-specific glycosylation analysis via LC/MS

The virus particles were collected from 60 to 80 mL of infectious allantoic fluid from chicken embryonated eggs via ultracentrifugation, followed by a 30% sucrose cushion ultracentrifugation to remove impurities. The pellet was dissolved in 100 µL of PBS with 0.08% sodium azide and then precipitated with acetone (final concentration of 80%) at −20°C overnight. After centrifugation at 13,000 rpm for 15 min, the precipitate was recovered and dissolved in 100 mM Tris-HCl (pH 9.0) containing 12 mM sodium deoxycholate and 12 mM sodium lauroylsarcosinate. The protein samples were treated with dithiothreitol (equal weight of protein, 37°C, 1 h) to reduce disulfide bonds and then alkylated with iodoacetamide (2.5 times the weight of protein, 37°C, 1 h, in the dark) and quenched with dithiothreitol (half the weight of protein, 37°C, 5 min). The proteins were then diluted fourfold with 50 mM ammonium bicarbonate and digested with rLys-C endopeptidase (Promega), followed by treatment with Trypsin Gold (Promega). An aliquot of each digest was treated with PNGase F (Takara Bio) in H₂^18^O to remove *N*-glycans and label glycosylated asparagines with the stable isotope ^18^O (isotope-coded glycosylation site-specific tagging [IGOT] method; (37, 38 ). The products were lyophilized, redissolved in nonlabeled water, treated with trypsin at 4°C overnight, and purified with MonoTip C18 (GL Sciences, Tokyo, Japan). These labeled peptides were subsequently analyzed using an LC/MS system with a nanoflow LC, UltiMate 3000 RSLCnano (Thermo Fisher Scientific), and an Orbitrap Eclipse Tribrid mass spectrometer (Thermo Fisher Scientific) as described previously for IGOT-LC/MS. Briefly, the peptides were separated using a C18 tip column (Aurora Ultimate, IonOpticks, Australia, 0.075 mm id ×25 cm length) at 300 nL/min with an acetonitrile gradient (3%–35% acetonitrile in 0.1% formic acid over 45 min). MS2 spectra were obtained data-dependently (positive mode, cycle time: 3 s, MS1 resolution: 120,000, range: 377–2000, analyzer: Orbitrap, maximum injection time: 100 ms, MS2 resolution: 60,000, range: 135–2000, isolation window: 1.6, analyzer: Orbitrap, activation: HCD, maximum injection time: 200 ms, normalized collision energy: stepped (20, 30, 40)). For site-specific glycosylation analyses, the MS2 spectra were searched using Mascot (ver. 2.8.0.1, Matrix Science, London, UK) with a UniProtKB sequence database (tax id: 9031, including HA sequences). Search conditions were as follows: method: MS/MS ion search, target FDR: 1%, enzyme: semi-trypsin/Lys-C, fixed modification: carbamidomethyl (C), variable modifications: ammonium loss (peptide N-term C), delta:H (1)N(−1)18O(1)(N), Gln >pyroGlu (peptide N-term Q), and Oxidation (M), mass tolerance (MS1): seven ppm, MS2: 10 ppm, max missed cleavage: 2. All spectra of the target peptides were manually inspected for correct peptide identification and site-specific glycosylation.

### Prediction of RNA secondary structure

The RNAfold web server, provided by the ViennaRNA web services (http://rna.tbi.univie.ac.at/; 33), and the CentroidFold web server (http://rtools.cbrc.jp/centroidfold/; 66) were used to predict the secondary structure of the RNA sequences of NA-stalk domains. The RNA sequences of partial NA segments (129 nt), including the NA-stalk coding regions, were submitted to either of the above web servers using the default parameters.

### Virus elution assay

The function of NA was evaluated by assessing the ability of viruses to elute agglutinated chicken erythrocytes, as previously described by Castrucci and Kawaoka (34). The viruses were adjusted to 32 HAU with PBS and serially diluted in borate-buffered saline with calcium (6.8 mM CaCl_2_, 154 mM NaCl in 20 mM borate buffer, pH 7.2) in technical triplicate. Then, 50 µL of each dilution was incubated with an equivalent volume of 0.5% chicken erythrocytes at 4°C for 1 h in a U-bottom plate (Corning Inc., Corning, NY, USA). The plate was then moved to 37°C, and the dissolution of hemagglutination due to NA activity was recorded periodically for 8 h. The elution activity was evaluated as the proportion of wells where hemagglutination was canceled relative to the total number of wells that had been hemagglutinated before incubation at 37°C.

### NA enzymatic activity

NA enzymatic activity was measured using 2′-(4-Methylumbelliferyl)–α–D–N-acetylneuraminic acid (MUNANA; Sigma-Aldrich) as a soluble substrate, as described previously (67). Briefly, the viruses were mixed with serially diluted MUNANA in 96-well black-bottomed plates. The mixture was exerted at 360 nm at 1 min intervals, and the emission signal was periodically monitored for 30 min at 37°C using POWER SCAN 4 (Agilent Technologies). The initial reaction rate under each condition was calculated and plotted to obtain the Michaelis constant, *K*_m_, using R software.

### Comparison of NA-stalk domain of N7 AIVs

A total of 1,498 amino acid sequences of N7 NA covering the stalk domain were obtained from the Global Initiative on Sharing All Influenza Data (GISAID, https://gisaid.org/) and aligned using multiple sequence alignment software GENETYX ver. 15 (Nihon Server). Seventy-seven sequences exhibited amino acid deletions in the stalk domain. Each pattern of deletion was categorized based on the original host of each virus as indicated in the strain names. The remaining 1,421 sequences were analyzed to identify amino acid sequences at potential *N*-glycosylation sites within the stalk.

## Data Availability

The data supporting the findings of this study are available within the article and its supplemental material. All sequence data of the rescued viruses are available in Tables S1 and S2.

## SUPPLEMENTAL MATERIAL

Supplemental figures jvi.01478-24-s0001.pdf Figures S1 to S16.

Table S1 jvi.01478-24-s0002.xlsx Recombinant viruses generated by reverse genetics.

Table S2 jvi.01478-24-s0003.xlsx Nucleotide sequences of all segments of recombinant viruses.

Table S3 jvi.01478-24-s0004.pdf Amino acid sequences of recovered viruses from six chickens inoculated with L4/P0NAΔG by sanger sequencing.

Table S4 jvi.01478-24-s0005.xlsx Amino acid sequences of low-frequent variants recovered from chickens inoculated with L4/P0ΔG detected by NGS.

Table S5 jvi.01478-24-s0006.pdf Amino acid sequences of viruses passaged in chicken embryo with L4/P0NAΔG.

ASM does not own the copyrights to Supplemental Material that may be linked to, or accessed through, an article. The authors have granted ASM a non-exclusive, world-wide license to publish the Supplemental Material files. Please contact the corresponding author directly for reuse.
