# Supplementary material for: Deglycosylation and truncation in the neuraminidase stalk are functionally equivalent in enhancing the pathogenicity of a high pathogenicity avian influenza virus in chickens
Source: J Virol. 2025 Feb 14;99(3):e01478-24. doi: 10.1128/jvi.01478-24 (PMC11915841; doi:10.1128/jvi.01478-24)
Supplement: Supplemental figures — Figures S1 to S16. [file jvi.01478-24-s0001.pdf]

Supplemental Figure legends

Supplemental Figure 1. RNA secondary structures predicted using RNAfold WebServer

(A) Minimum free energy (MFE) structure. (B) Centroid structure. Both structures were generated using default settings from the RNAfold WebServer (http://rna.tbi.univie.ac.at//cgi-bin/RNAWebSuite/RNAfold.cgi).

Supplemental Figure 2. RNA secondary structures predicted using CentroidFold

RNA secondary structures were predicted using CentroidFold (http://rtools.cbrc.jp/cgi-bin/index.cgi) with the default McCaskill (BL) model.

Supplemental Figure 3. Gene construction for the FLAG-fused NA protein and comparison of NA intake in virus virions among Vac2 mutants

(A) Schematic representation of the gene construction for generating the FLAG (DYKDDDDK)-fused NA protein. (B) NA intake in the virus virions of Vac2 mutants was assessed by comparing NA/NP ratios of the virus virions immunoprecipitated with an anti-HA antibody. The relative intensity of three spots was calculated and normalized to Vac2/P0NA.

Supplemental Figures 4–7. MS2 spectra and mass chromatograms of the peptide of P0NA with three or four glycans to identify site occupancy

Supplemental Figures 8–12. MS2 spectra and mass chromatograms of the peptide of P0NA-Y65H with three or four glycans to identify site occupancy

Supplemental Figure 13. MS2 spectrum and mass chromatograms of the peptide of P0NAΔGlyco-Q47N to identify site occupancy

Supplemental Figures 14–16. MS2 spectra and mass chromatograms of the peptide of P0NAΔGlyco-Q56,57N with one or two glycans to identify site occupancy

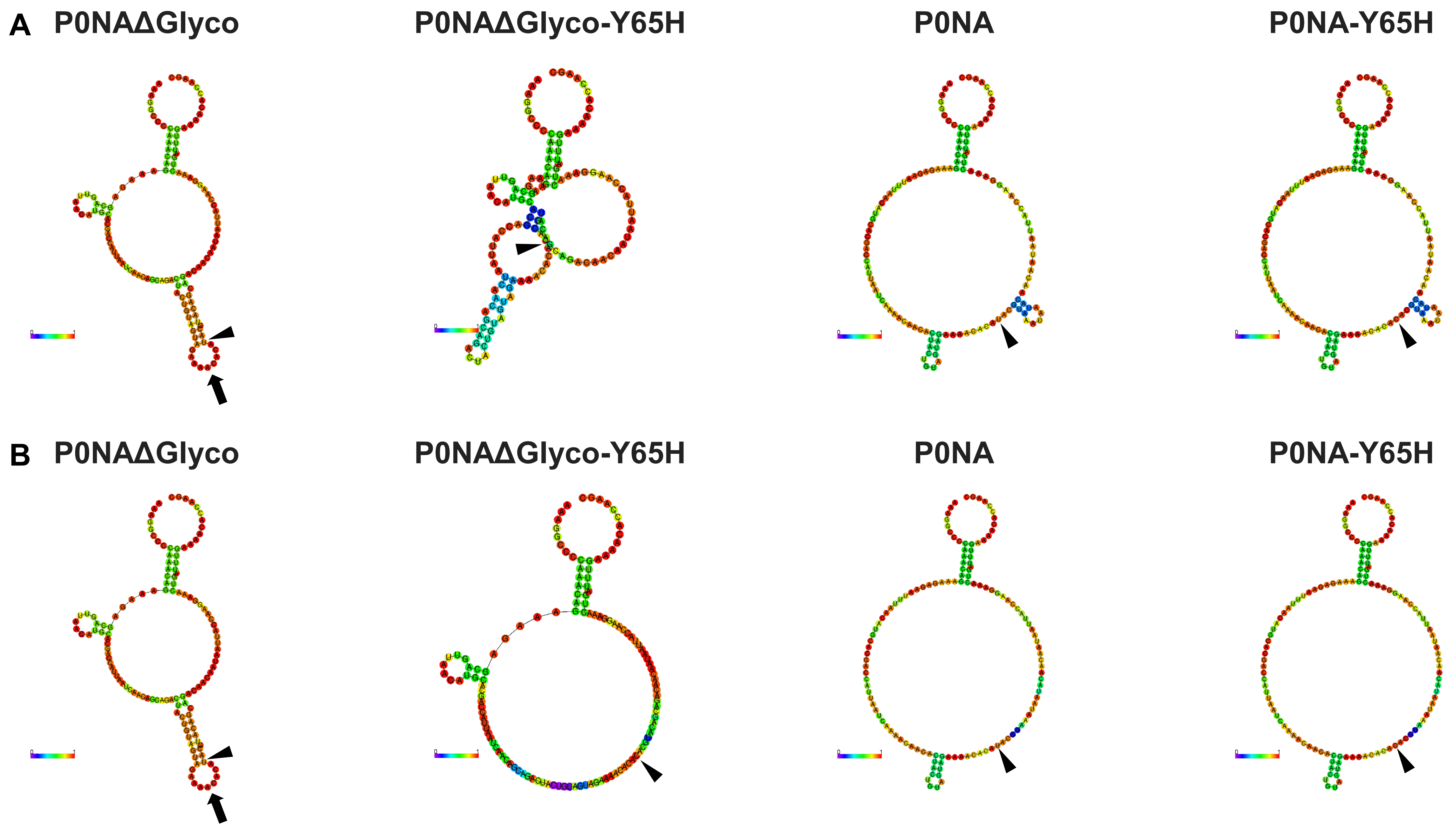

Supplemental Figure 1

Kobayashi *et al*

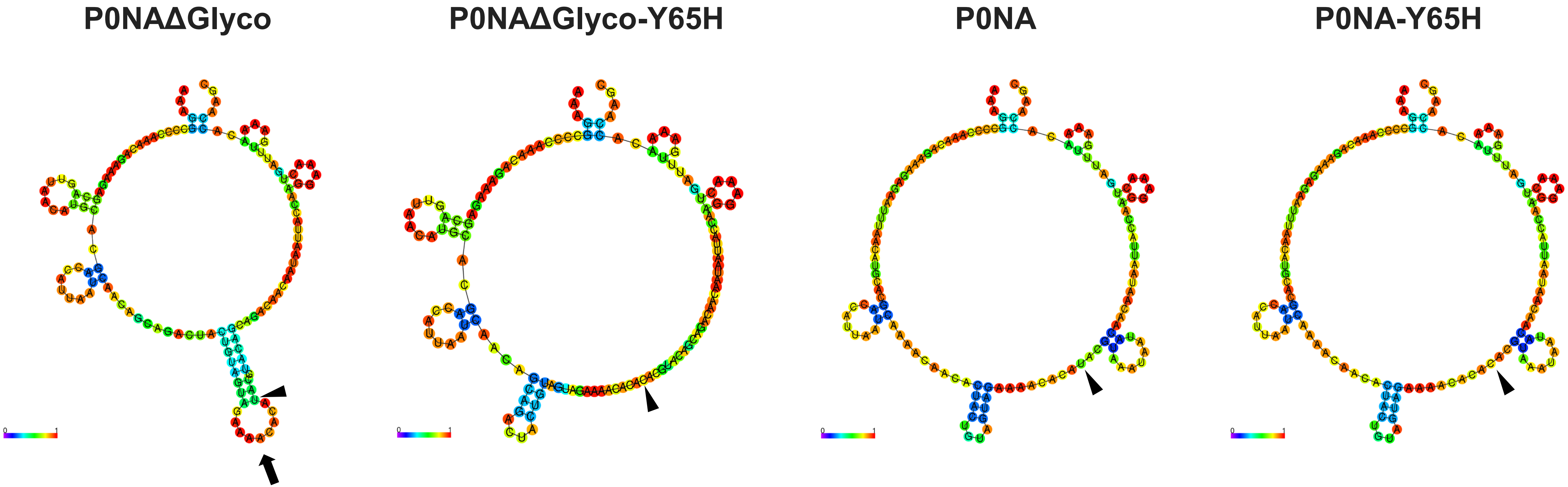

Supplemental Figure 2

Kobayashi *et al*

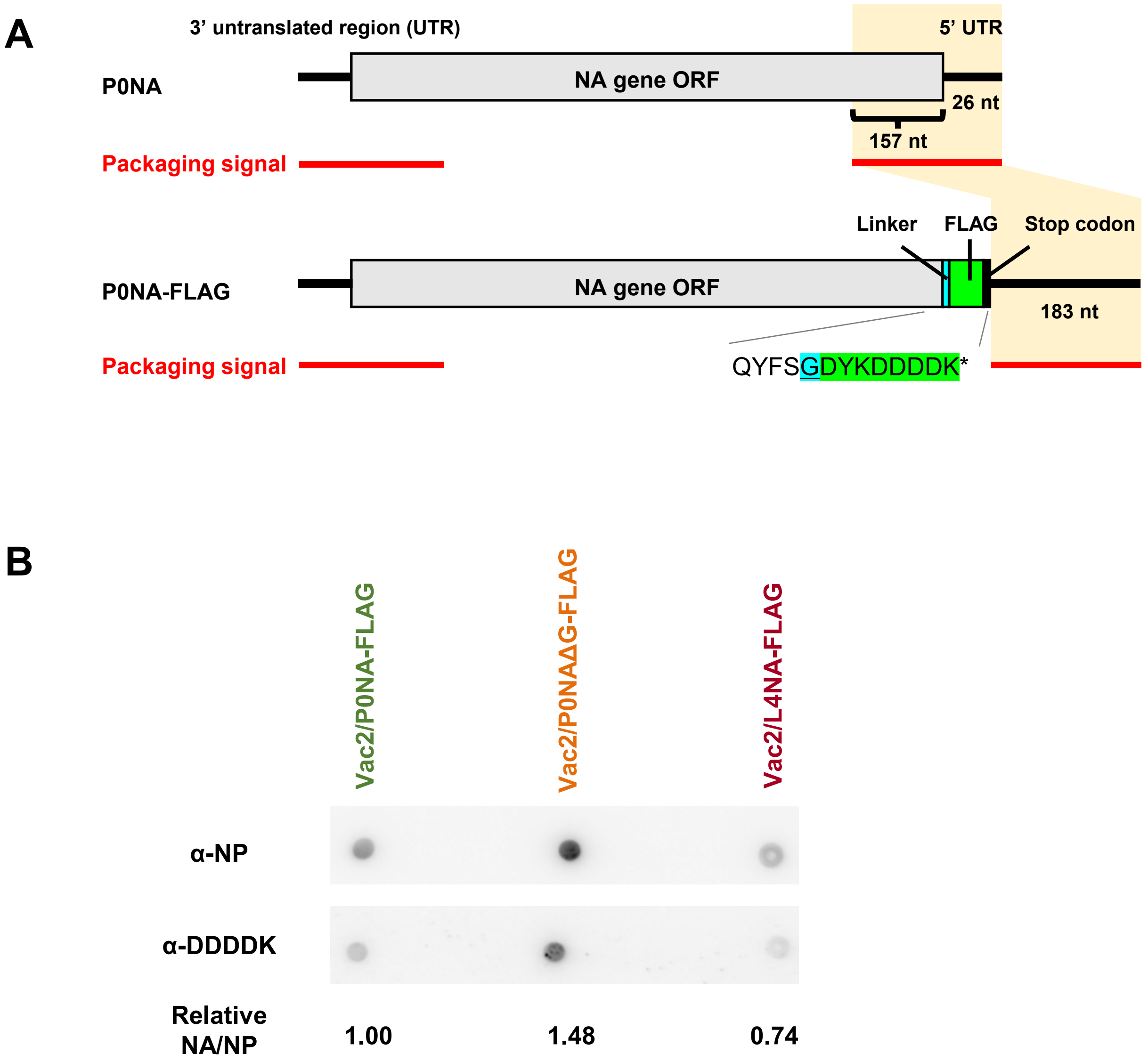

Supplemental Figure 3 Kobayashi *et al*

A

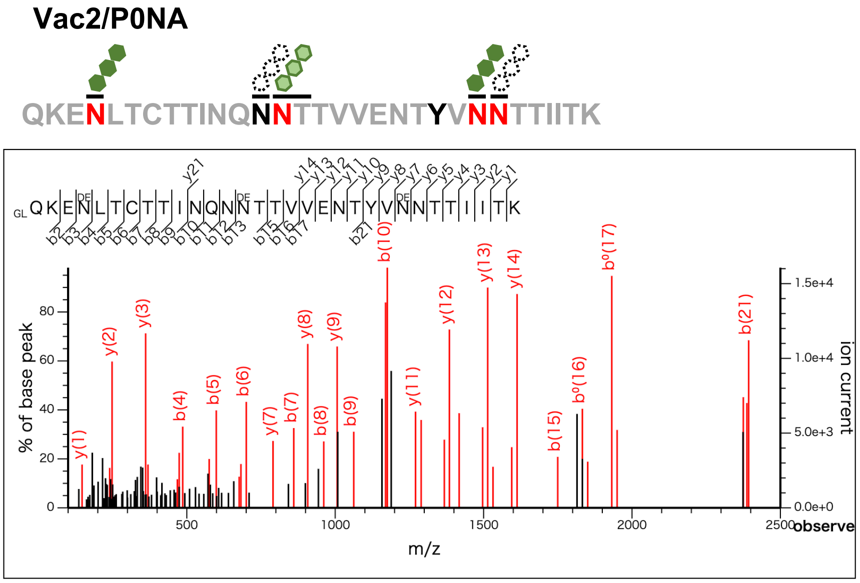

N-glycosylation site was occupied

N-glycosylation sites which are placed in the MS2 fragment could be occupied, while the possibility of modification in the other amino acids could not be denied

MS2 fragment included double NNTT sequons and either of two sites was occupied

N-glycosylation site was not occupied

B

#	b	Seq.	y	#
1	112.0393	Q	—	31
2	240.1343	K	3450.6814	30
3	369.1769	E	3322.5864	29
4	486.2080	N^DE	3193.5438	28
5	599.2921	L	3076.5126	27
6	700.3398	T	2963.4286	26
7	860.3704	C	2862.3809	25
8	961.4181	T	2702.3503	24
9	1062.4658	T	2601.3026	23
10	1175.5499	I	2500.2549	22
11	1289.5928	N	2387.1708	21
12	1417.6514	Q	2273.1279	20
13	1531.6943	N	2145.0693	19
14	1648.7255	N^DE	2031.0264	18
15	1749.7732	T	1913.9952	17
16	1850.8208	T	1812.9475	16
17	1949.8893	V	1711.8999	15
18	2048.9577	V	1612.8314	14
19	2178.0003	E	1513.7630	13
20	2292.0432	N	1384.7204	12
21	2393.0909	T	1270.6775	11
22	2556.1542	Y	1169.6298	10
23	2655.2226	V	1006.5665	9
24	2772.2538	N^DE	907.4981	8
25	2886.2967	N	790.4661	7
26	2987.3444	T	676.4240	6
27	3088.3921	T	575.3763	5
28	3201.4761	I	474.3286	4
29	3314.5602	I	361.2445	3
30	3415.6079	T	248.1605	2
31	—	K	147.1128	1

C

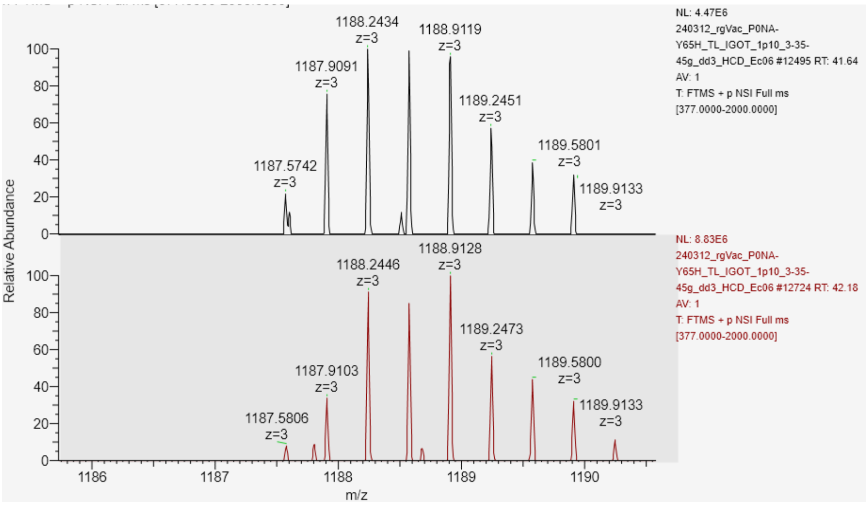

Supplemental Figure 4 **Kobayashi *et al***

A

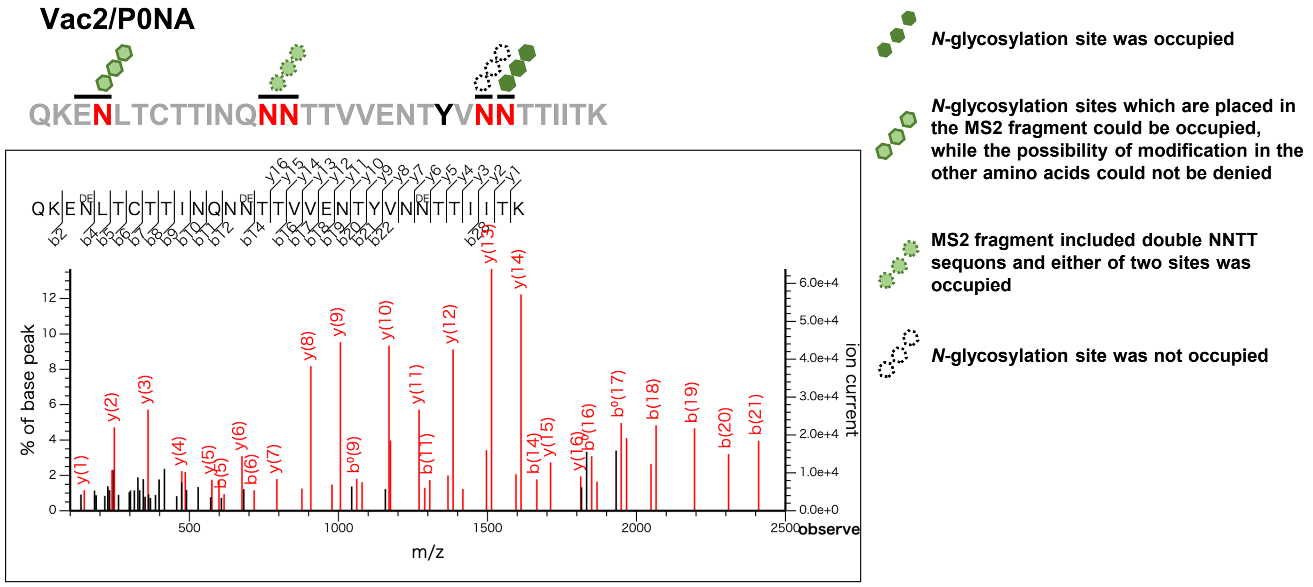

B

#	b	Seq.	y	#
1	129.0659	Q	—	31
2	257.1608	K	3450.6814	30
3	386.2034	E	3322.5864	29
4	503.2346	N^{DE}	3193.5438	28
5	616.3181	L	3076.5126	27
6	717.3663	T	2963.4286	26
7	877.3970	C	2862.3809	25
8	978.4441	T	2702.3503	24
9	1079.4923	T	2601.3026	23
10	1192.5764	I	2500.2549	22
11	1306.6193	N	2387.1708	21
12	1434.6779	Q	2273.1279	20
13	1548.7208	N	2145.0693	19
14	1665.7520	N^{DE}	2031.0264	18
15	1766.7997	T	1913.9952	17
16	1867.8474	T	1812.9475	16
17	1966.9158	V	1711.8999	15
18	2065.9842	V	1612.8314	14
19	2195.0268	E	1513.7630	13
20	2309.0691	N	1384.7204	12
21	2410.1174	T	1270.6775	11
22	2573.1807	Y	1169.6298	10
23	2672.2492	V	1006.5665	9
24	2786.2921	N	907.4981	8
25	2903.3233	N^{DE}	793.4552	7
26	3004.3710	T	676.4240	6
27	3105.4186	T	575.3763	5
28	3218.5027	I	474.3286	4
29	3331.5868	I	361.2445	3
30	3432.6344	T	248.1605	2
31	—	K	147.1128	1

C

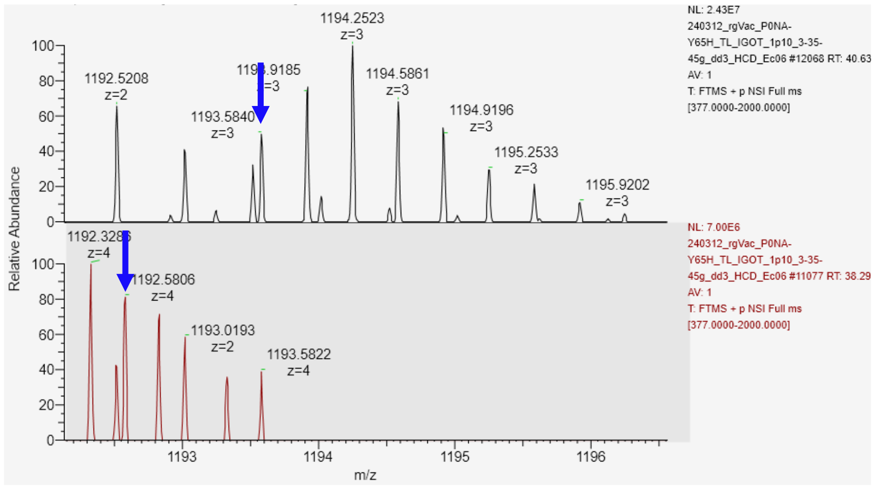

D

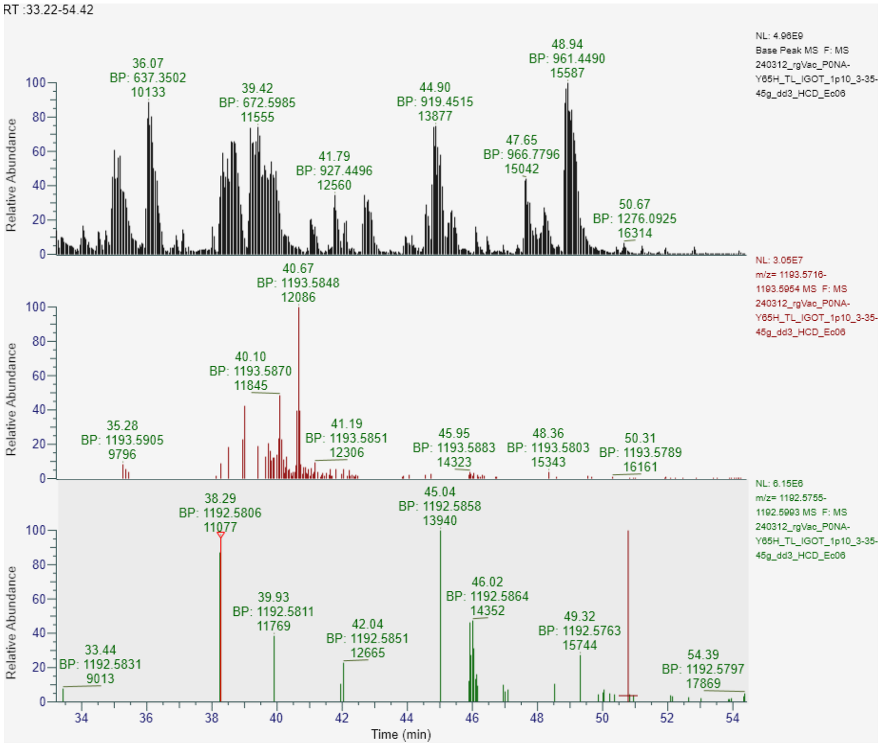

Supplemental Figure 5

Kobayashi *et al*

A

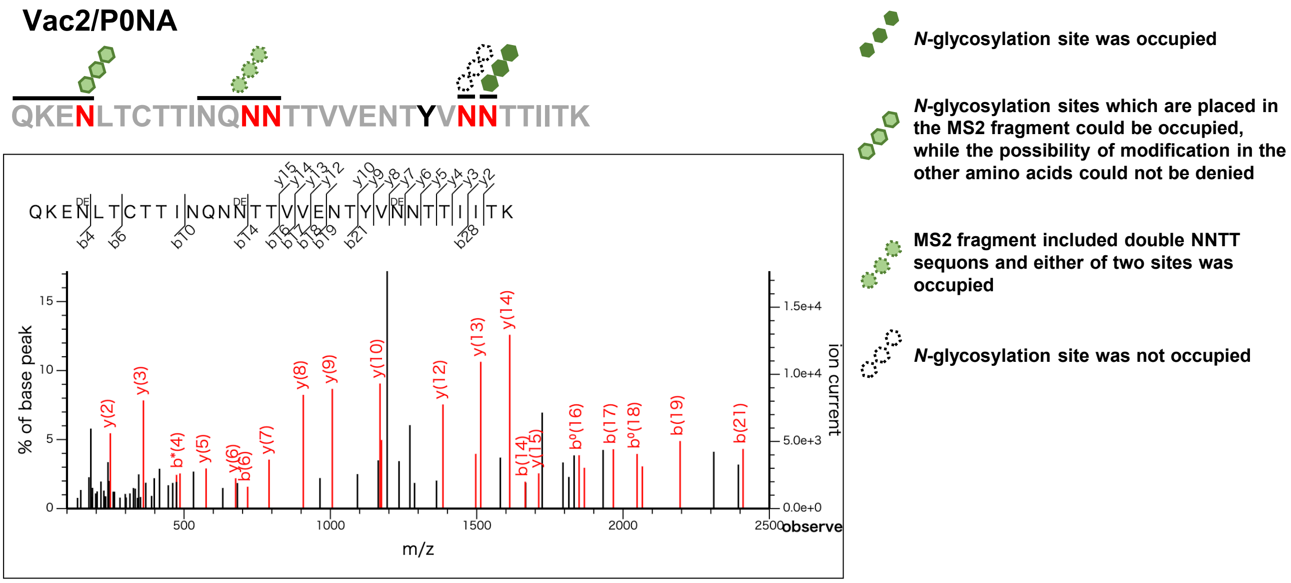

B

#	b	Seq.	y	#
1	129.0659	Q	—	31
2	257.1608	K	3450.6814	30
3	386.2034	E	3322.5864	29
4	503.2346	N^{DE}	3193.5438	28
5	616.3187	L	3076.5126	27
6	**717.3663**	T	2963.4286	26
7	877.3970	C	2862.3809	25
8	978.4447	T	2702.3503	24
9	1079.7923	T	2601.3026	23
10	1192.5764	I	2500.2549	22
11	1306.6193	N	2387.1708	21
12	1434.6779	Q	2273.1279	20
13	1548.7208	N	2145.0693	19
14	**1665.7520**	N^{DE}	2031.0264	18
15	1766.7997	T	1913.9952	17
16	**1867.8474**	T	1812.9475	16
17	**1966.9158**	V	**1711.8999**	15
18	**2065.9842**	V	**1612.8314**	14
19	**2195.0268**	E	**1513.7630**	13
20	2309.0697	N	**1384.7204**	12
21	**2410.1174**	T	1270.6775	11
22	2573.1807	Y	**1169.6298**	10
23	2672.2492	V	**1006.5665**	9
24	2789.2803	N^{DE}	**907.4981**	8
25	2903.3233	N	**790.4669**	7
26	3004.3710	T	**676.4240**	6
27	3105.4186	T	**575.3763**	5
28	**3218.5027**	I	**474.3286**	4
29	3331.5868	I	**361.2445**	3
30	3432.6344	T	**248.1605**	2
31	—	K	147.1128	1

C

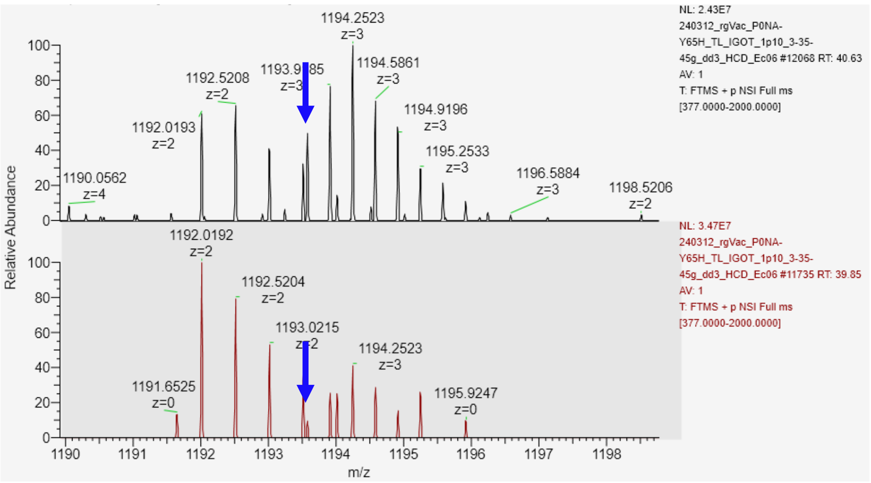

Supplemental Figure 6 **Kobayashi *et al***

A

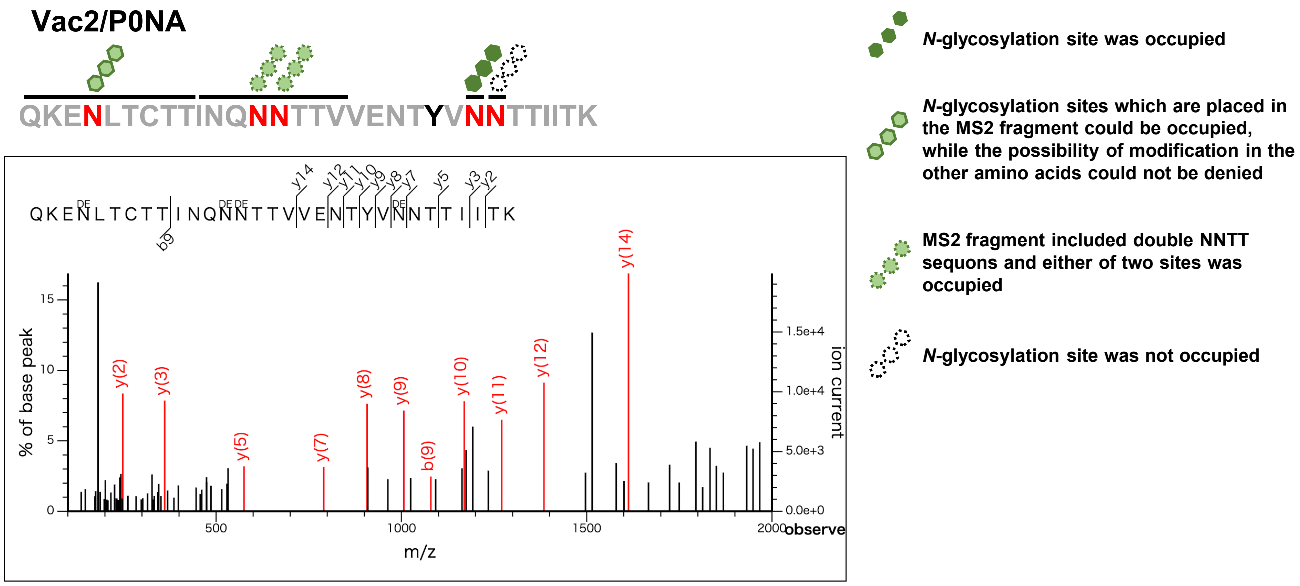

B

#	b	Seq.	y	#
1	129.0659	Q	—	31
2	257.1608	K	3453.6697	30
3	386.2034	E	3325.5747	29
4	503.2346	N^DE	3196.5321	28
5	616.3187	L	3079.5009	27
6	717.3663	T	2966.4168	26
7	877.3970	C	2865.3692	25
8	978.4447	T	2705.3385	24
9	**1079.4923**	T	2604.2908	23
10	1192.5764	I	2503.2432	22
11	1306.6193	N	2390.1591	21
12	1434.6779	Q	2276.1162	20
13	1551.7091	N^DE	2148.0576	19
14	1668.7403	N^DE	2031.0264	18
15	1769.7880	T	1913.9952	17
16	1870.8357	T	1812.9475	16
17	1969.9041	V	1711.8999	15
18	2068.9725	V	**1612.8314**	14
19	2198.0151	E	1513.7630	13
20	2312.0580	N	**1384.7204**	12
21	2413.1057	T	**1270.6775**	11
22	2576.1690	Y	**1169.6298**	10
23	2675.2374	V	**1006.5665**	9
24	2792.2686	N^DE	**907.4981**	8
25	2906.3115	N	**790.4669**	7
26	3007.3592	T	676.4240	6
27	3108.4069	T	**575.3763**	5
28	3221.4910	I	474.3286	4
29	3334.5750	I	**361.2445**	3
30	3435.6227	T	**248.1605**	2
31	—	K	147.1128	1

C

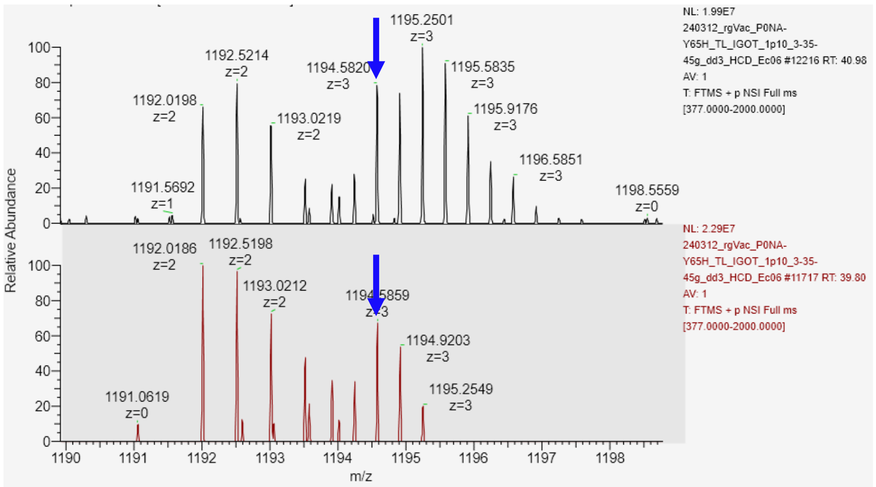

Supplemental Figure 7 **Kobayashi *et al***

A Vac2/P0NA-65H

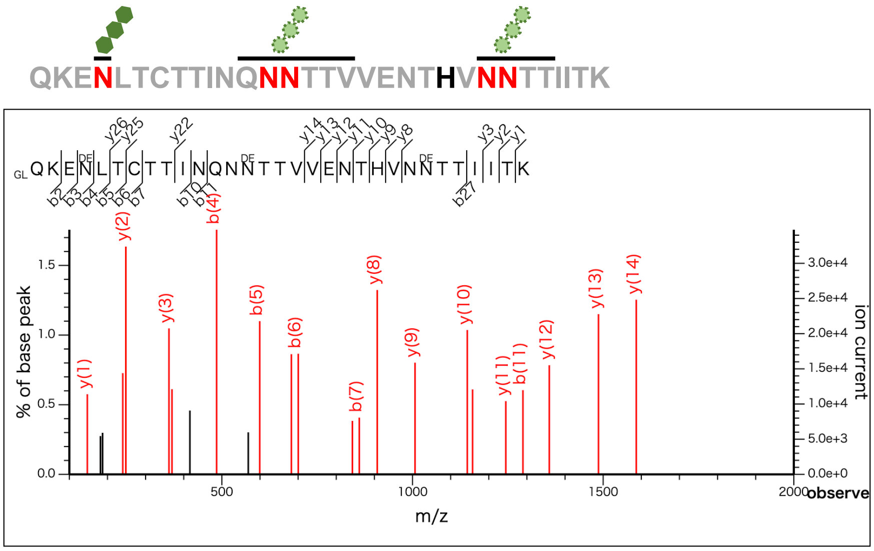

N-glycosylation site was occupied

N-glycosylation sites which are placed in the MS2 fragment could be occupied, while the possibility of modification in the other amino acids could not be denied

MS2 fragment included double NNTT sequons and either of two sites was occupied

N-glycosylation site was not occupied

B

#	b	Seq.	y	#
1	112.0393	Q	—	31
2	240.1343	K	3424.6770	30
3	369.1769	E	3296.5820	29
4	486.2080	N^{DE}	3167.5394	28
5	599.2921	L	3050.5082	27
6	700.3398	T	2937.4242	26
7	860.3704	C	2836.3765	25
8	961.4181	T	2676.3458	24
9	1062.4658	T	2575.2982	23
10	1175.5499	I	2474.2505	22
11	1289.5928	N	2361.1664	21
12	1417.6514	Q	2247.1235	20
13	1531.6943	N	2119.0649	19
14	1648.7255	N^{DE}	2005.0220	18
15	1749.7732	T	1887.9908	17
16	1850.8208	T	1786.9431	16
17	1949.8893	V	1685.8954	15
18	2048.9577	V	1586.8270	14
19	2178.0003	E	1487.7586	13
20	2292.0432	N	1358.7160	12
21	2393.0909	T	1244.6731	11
22	2530.1498	Y	1143.6254	10
23	2629.2182	V	1006.5665	9
24	2743.2611	N	907.4981	8
25	2860.2923	N^{DE}	793.4552	7
26	2961.3400	T	676.4240	6
27	3062.3877	T	575.3763	5
28	3175.4717	I	474.3286	4
29	3288.5558	I	361.2445	3
30	3389.6035	T	248.1605	2
31	—	K	147.1128	1

C

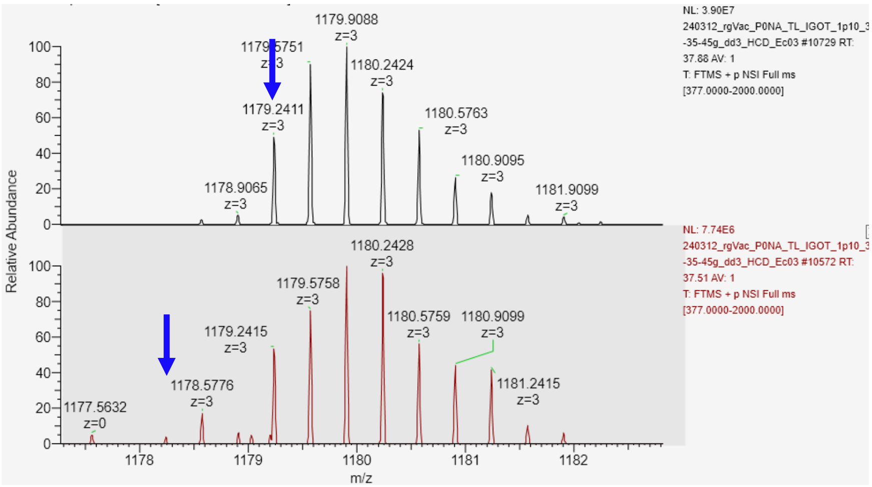

D

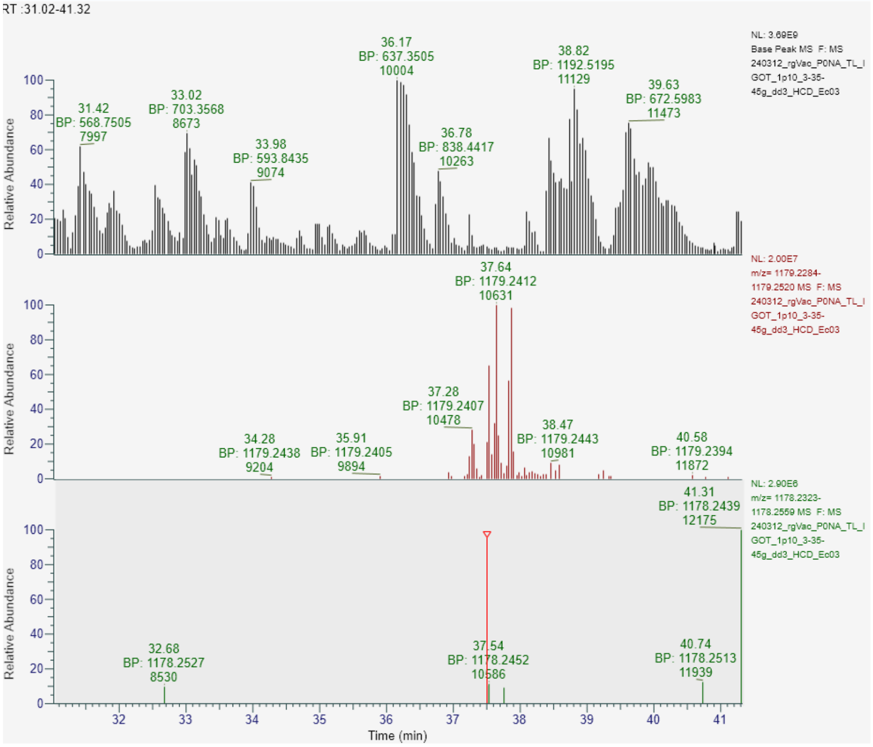

Supplemental Figure 8 **Kobayashi *et al***

A

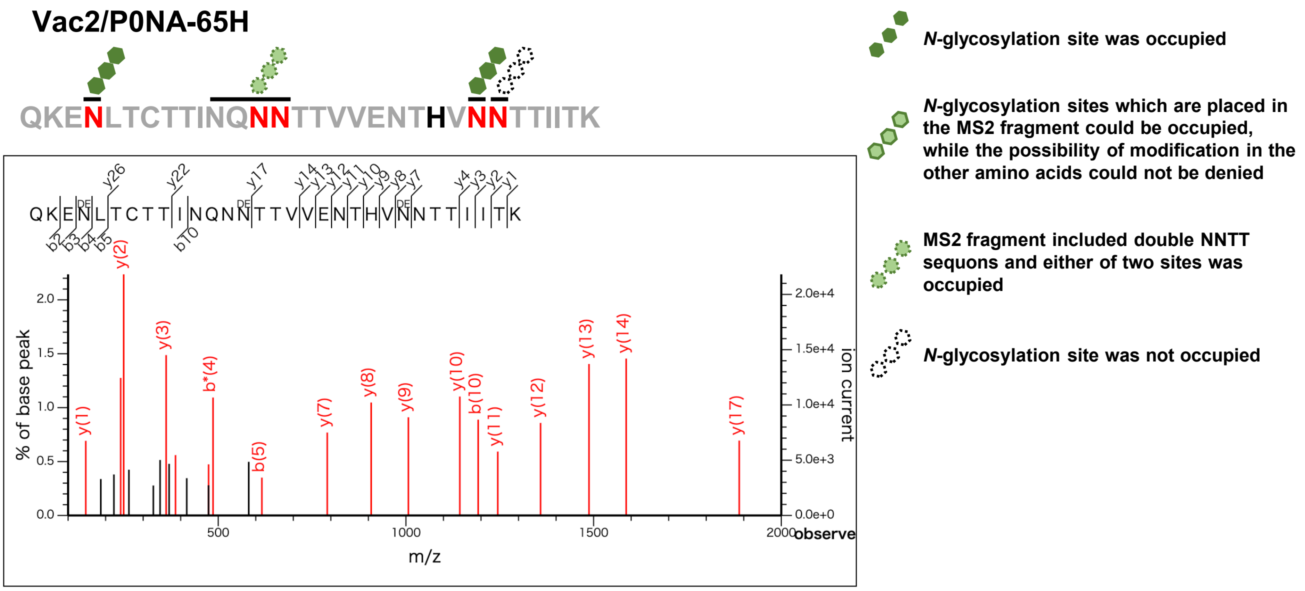

B

#	b	Seq.	y	#
1	129.0659	Q	—	31
2	257.1608	K	3424.6770	30
3	386.2034	E	3296.5820	29
4	503.2346	N[DE]	3167.5394	28
5	616.3187	L	3050.5082	27
6	717.3663	T	2937.4242	26
7	877.3970	C	2836.3765	25
8	978.4447	T	2676.3458	24
9	1079.4923	T	2575.2982	23
10	1192.5764	I	2474.2505	22
11	1306.6193	N	2361.1664	21
12	1434.6779	Q	2247.1235	20
13	1548.7208	N	2119.0649	19
14	1665.7520	N[DE]	2005.0220	18
15	1766.7997	T	1887.9908	17
16	1867.8474	T	1786.9431	16
17	1966.9158	V	1685.8954	15
18	2065.9842	V	1586.8270	14
19	2195.0268	E	1487.7586	13
20	2309.0697	N	1358.7160	12
21	2410.1174	T	1244.6731	11
22	2547.1763	Y	1143.6254	10
23	2646.2447	V	1006.5665	9
24	2763.2759	N[DE]	907.4981	8
25	2877.3189	N	790.4669	7
26	2978.3665	T	676.4240	6
27	3079.4142	T	575.3763	5
28	3192.4983	I	474.3286	4
29	3305.5823	I	361.2445	3
30	3406.6300	T	258.1605	2
31	—	K	147.1128	1

C

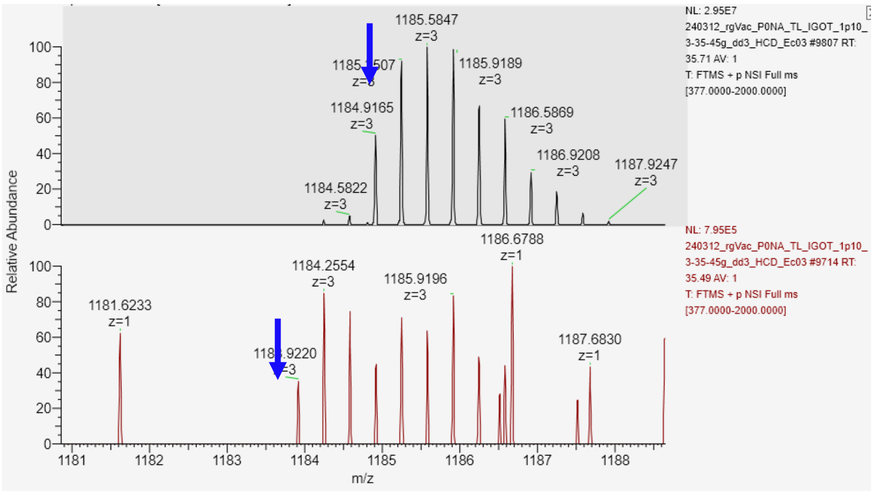

D

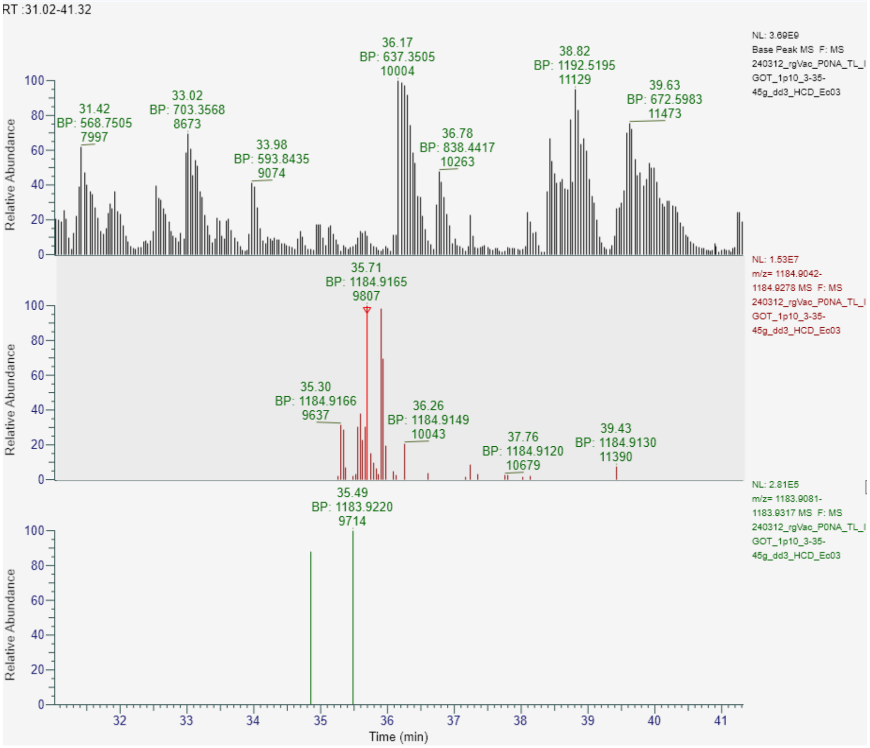

Supplemental Figure 9 **Kobayashi *et al***

A

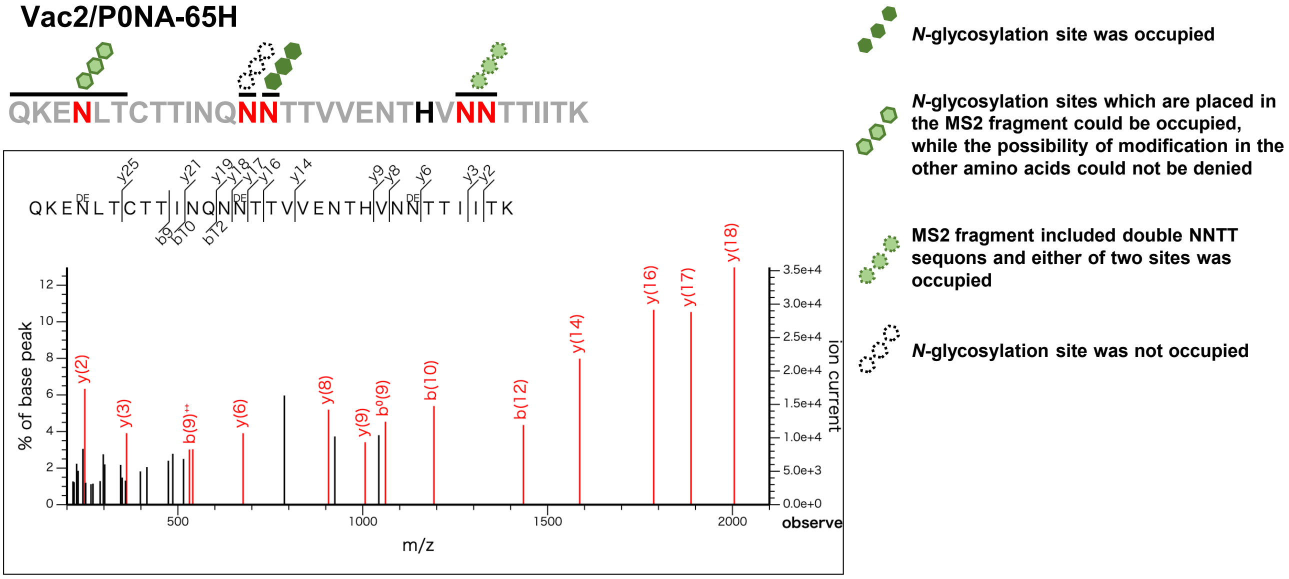

B

#	b	Seq.	y	#
1	129.0659	Q	—	31
2	257.1608	K	3424.6770	30
3	386.2034	E	3296.5820	29
4	503.2346	N^{DE}	3167.5394	28
5	616.3187	L	3050.5082	27
6	717.3663	T	2937.4242	26
7	877.3970	C	**2836.3765**	25
8	978.4447	T	2676.3458	24
9	1079.4923	T	2575.2982	23
10	**1192.5764**	I	2474.2505	22
11	1306.6193	N	**2361.1664**	21
12	**1434.6779**	Q	2247.1235	20
13	1548.7208	N	**2119.0649**	19
14	1665.7520	N^{DE}	**2005.0220**	18
15	1766.7997	T	**1887.9908**	17
16	1867.8474	T	**1786.9431**	16
17	1966.9158	V	1685.8954	15
18	2065.9842	V	**1586.8270**	14
19	2195.0268	E	1487.7586	13
20	2309.0697	N	1358.7160	12
21	2410.1174	T	1244.6731	11
22	2547.1763	Y	1143.6254	10
23	2646.2447	V	**1006.5665**	9
24	2760.2877	N	**907.4981**	8
25	2877.3189	N^{DE}	790.4669	7
26	2978.3665	T	**676.4240**	6
27	3079.4142	T	575.3763	5
28	3192.4983	I	474.3286	4
29	3305.5823	I	**361.2445**	3
30	3406.6300	T	**258.1605**	2
31	—	K	147.1128	1

C

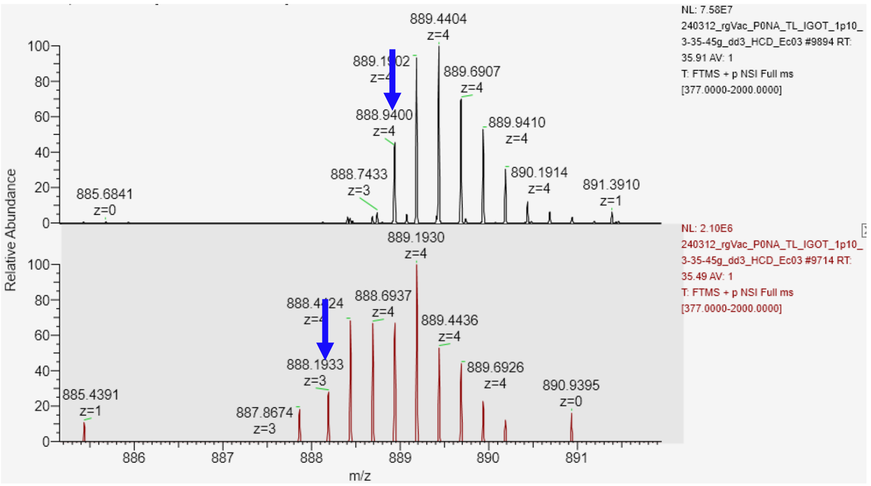

D

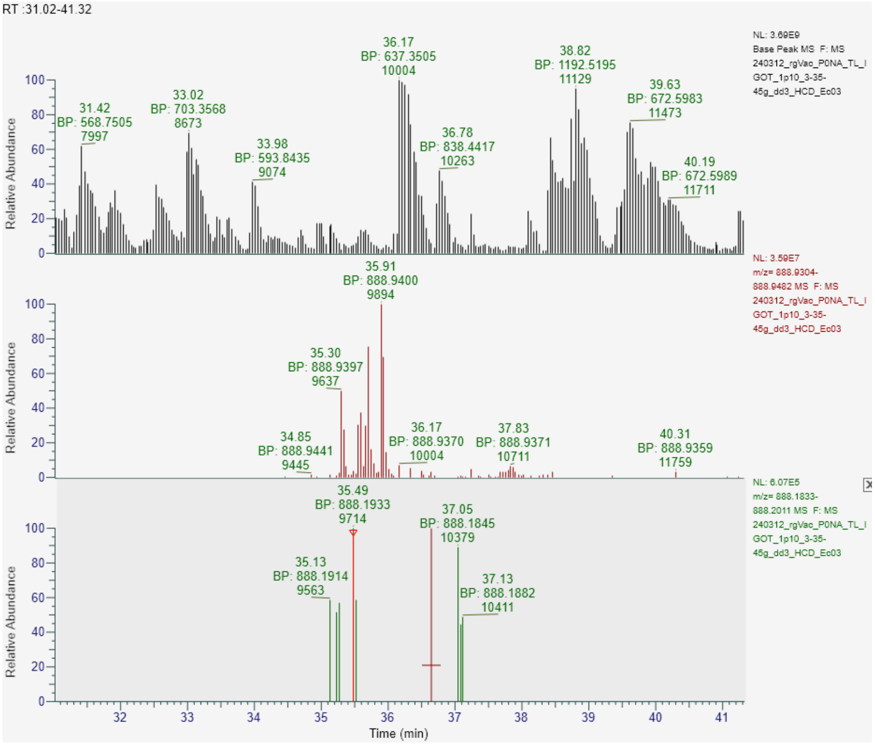

Supplemental Figure 10

Kobayashi *et al*

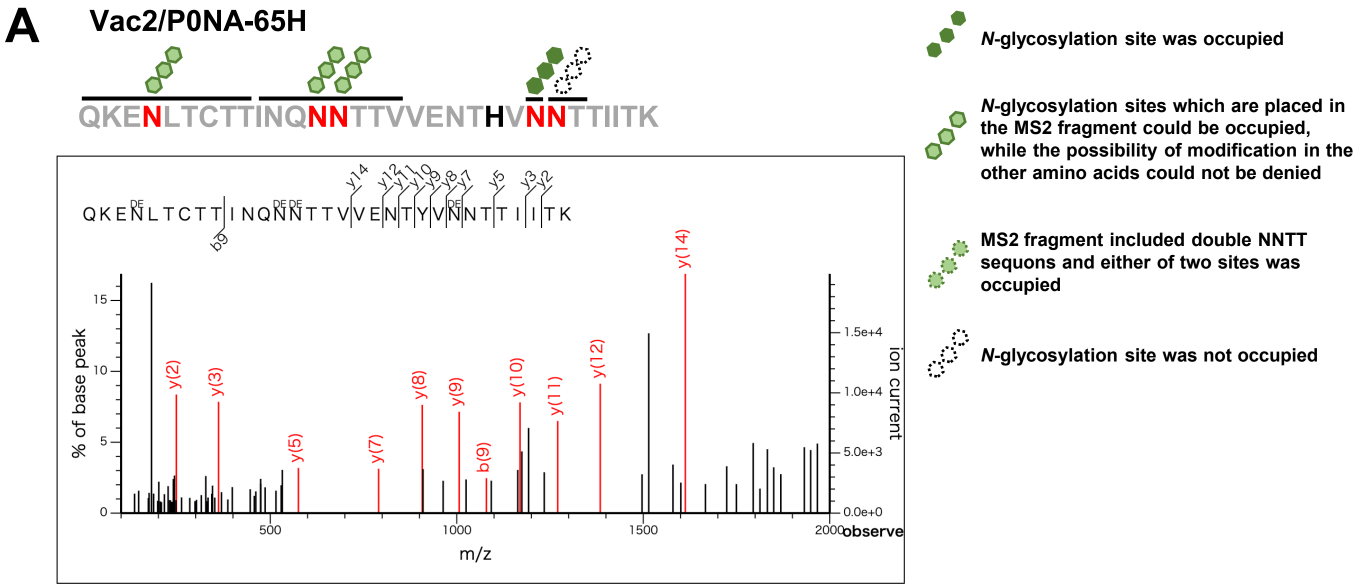

B

#	b	Seq.	y	#
1	129.0659	Q	—	31
2	257.1608	K	3427.6652	30
3	386.2034	E	3299.5703	29
4	503.2346	N^{DE}	3170.5277	28
5	616.3187	L	3053.4965	27
6	717.3663	T	2940.4124	26
7	877.3970	C	2839.3647	25
8	978.4447	T	2679.3341	24
9	1079.4923	T	2578.2864	23
10	1192.5764	I	2477.2387	22
11	1306.6193	N	2364.1547	21
12	1434.6779	Q	2250.1117	20
13	1548.7208	N^{DE}	2122.0532	19
14	1665.7520	N^{DE}	2008.0102	18
15	1766.7997	T	1890.9791	17
16	1867.8474	T	1789.9314	16
17	1966.9158	V	1688.8837	15
18	2065.9842	V	1589.8153	14
19	2195.0268	E	1490.7469	13
20	2309.0697	N	1361.7043	12
21	2410.1174	T	1247.6613	11
22	2547.1763	Y	1146.6137	10
23	2646.2447	V	1009.5548	9
24	2763.2759	N^{DE}	910.4863	8
25	2880.3071	N	793.4552	7
26	2981.3548	T	676.4240	6
27	3082.4025	T	575.3763	5
28	3195.4865	I	474.3286	4
29	3308.5706	I	361.2445	3
30	3409.6183	T	248.1605	2
31	—	K	147.1128	1

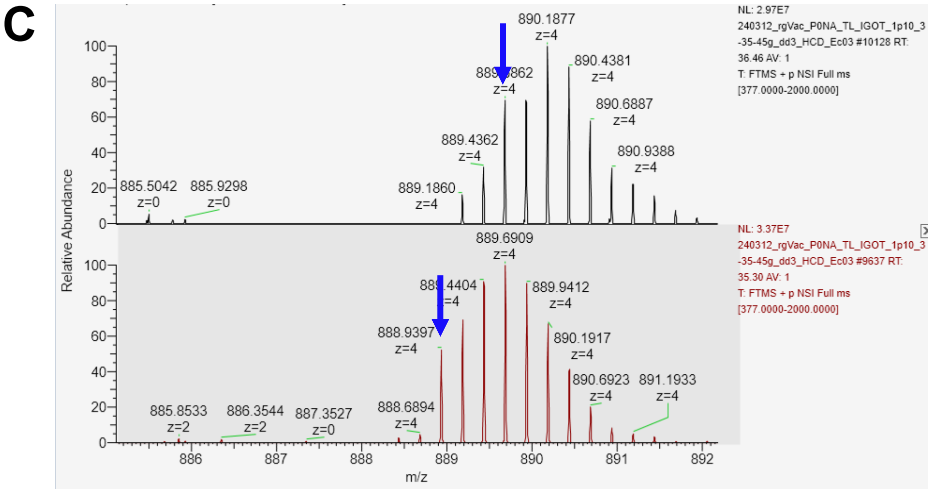

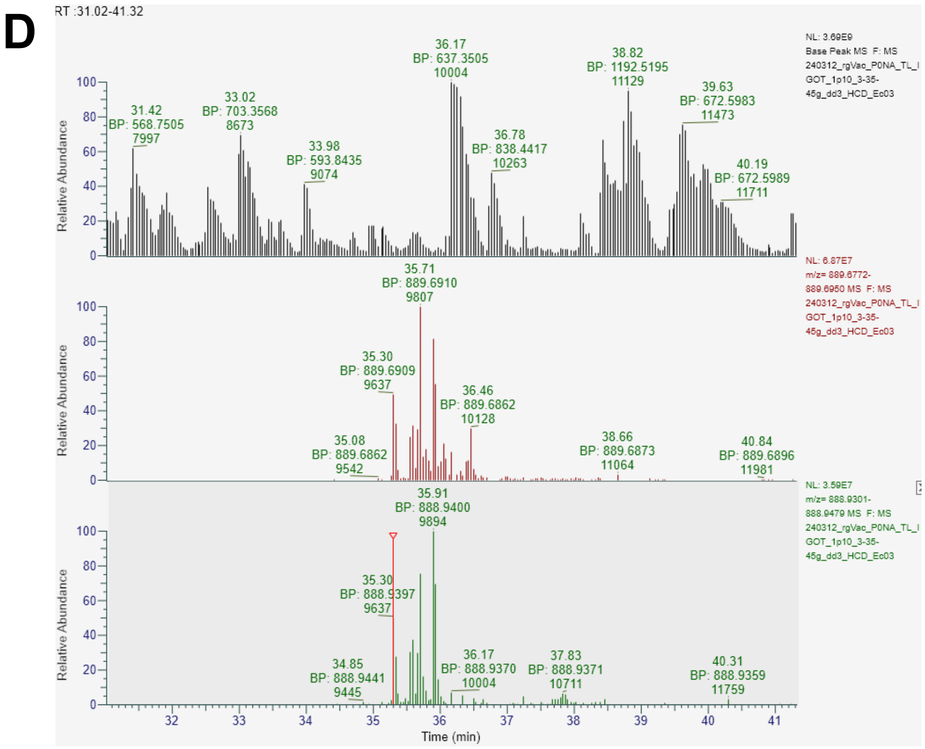

Supplemental Figure 11 **Kobayashi *et al***

A

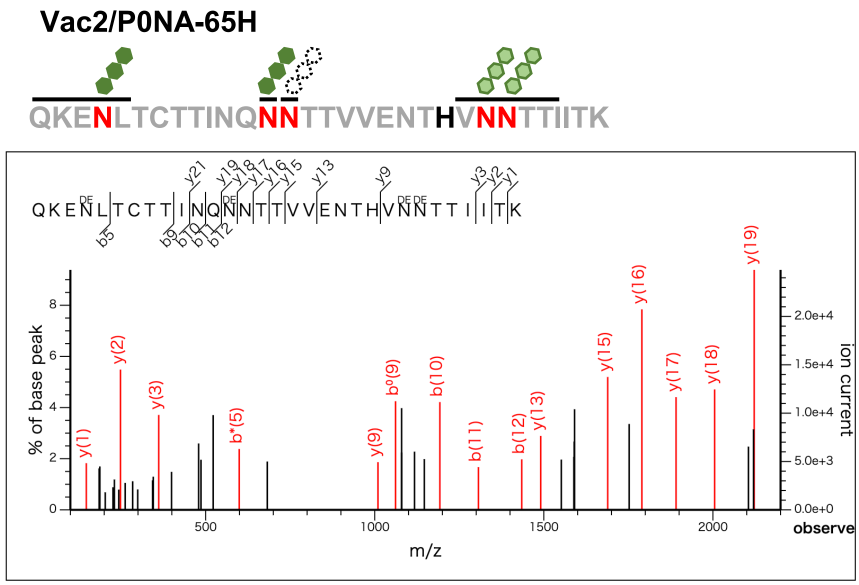

N-glycosylation site was occupied

N-glycosylation sites which are placed in the MS2 fragment could be occupied, while the possibility of modification in the other amino acids could not be denied

MS2 fragment included double NNTT sequons and either of two sites was occupied

N-glycosylation site was not occupied

B

#	b	Seq.	y	#
1	129.0659	Q	—	31
2	257.1608	K	3427.6652	30
3	386.2034	E	3299.5703	29
4	503.2346	N^DE	3170.5277	28
5	616.3187	L	3053.4965	27
6	717.3663	T	2940.4124	26
7	877.3970	C	2839.3647	25
8	978.4447	T	2679.3341	24
9	1079.4923	T	2578.2864	23
10	1192.5764	I	2477.2387	22
11	1306.6193	N	2364.1547	21
12	1434.6779	Q	2250.1117	20
13	1551.7091	N^DE	2122.0532	19
14	1665.7520	N	2005.0220	18
15	1766.7997	T	1890.9791	17
16	1867.8474	T	1789.9314	16
17	1966.9158	V	1688.8837	15
18	2065.9842	V	1589.8153	14
19	2195.0268	E	1490.7469	13
20	2309.0697	N	1361.7043	12
21	2410.1174	T	1247.6613	11
22	2547.1763	Y	1146.6137	10
23	2646.2447	V	1009.5548	9
24	2763.2759	N^DE	910.4863	8
25	2880.3071	N^DE	793.4552	7
26	2981.3548	T	676.4240	6
27	3082.4025	T	575.3763	5
28	3195.4865	I	474.3286	4
29	3308.5706	I	361.2445	3
30	3409.6183	T	248.1605	2
31	—	K	147.1128	1

C

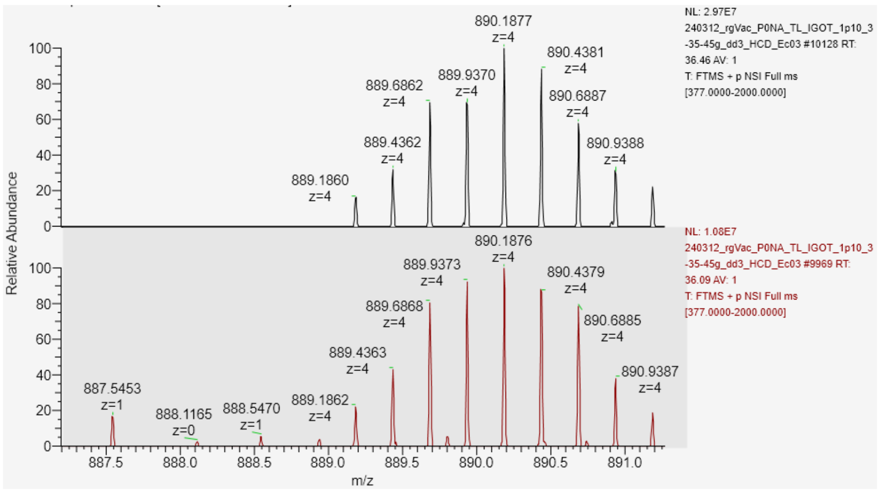

A

Vac2/P0NA-47N

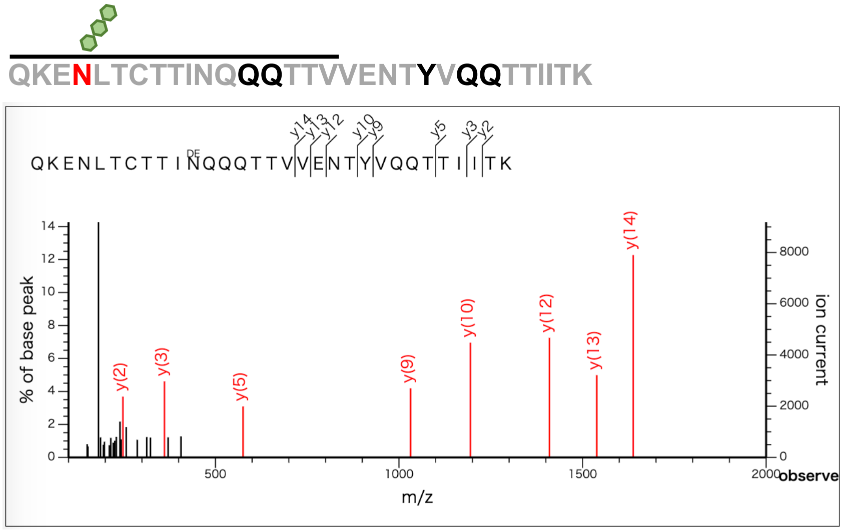

N-glycosylation site was occupied

N-glycosylation sites which are placed in the MS2 fragment could be occupied, while the possibility of modification in the other amino acids could not be denied

MS2 fragment included double NNTT sequons and either of two sites was occupied

N-glycosylation site was not occupied

B

#	b	Seq.	y	#
1	129.0659	Q	—	31
2	257.1608	K	3500.7675	30
3	386.2034	E	3372.6725	29
4	500.2463	N^DE	3243.6299	28
5	613.3304	L	3129.5870	27
6	714.3781	T	3016.5029	26
7	874.4087	C	2915.4552	25
8	975.4564	T	2755.4246	24
9	1076.5041	T	2654.3769	23
10	1189.5882	I	2553.3292	22
11	1306.6193	N	2440.2452	21
12	1434.6779	Q	2323.2140	20
13	1562.7365	Q	2195.1554	19
14	1690.7951	Q	2067.0968	18
15	1791.8428	T	1939.0383	17
16	1892.8904	T	1837.9906	16
17	1991.9588	V	1736.9429	15
18	2091.0273	V	1637.8745	14
19	2220.0699	E	1538.8061	13
20	2334.1128	N	1409.7635	12
21	2435.1605	T	1295.7205	11
22	2598.2238	Y	1194.6729	10
23	2697.2922	V	1031.6095	9
24	2825.3508	Q	932.5411	8
25	2953.4094	Q	804.4825	7
26	3054.4570	T	676.4240	6
27	3155.5047	T	575.3763	5
28	3268.5888	I	474.3286	4
29	3381.6728	I	361.2445	3
30	3482.7205	T	248.1605	2
31	—	K	147.1128	1

C

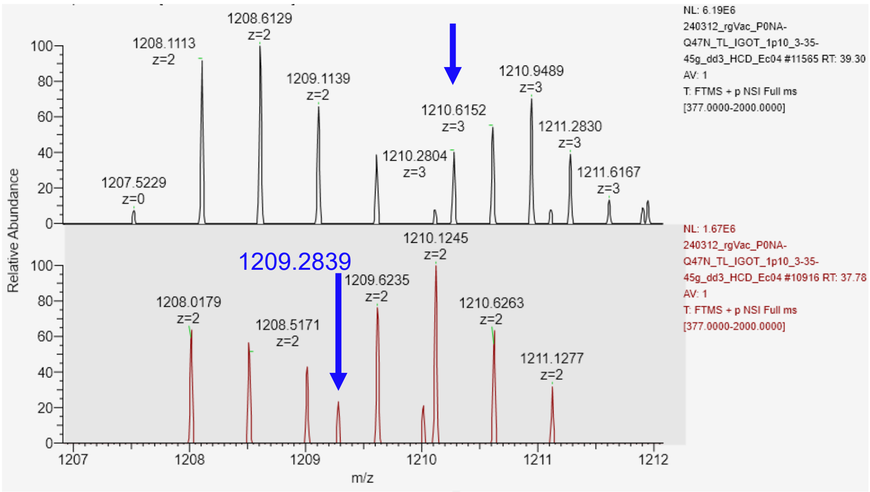

D

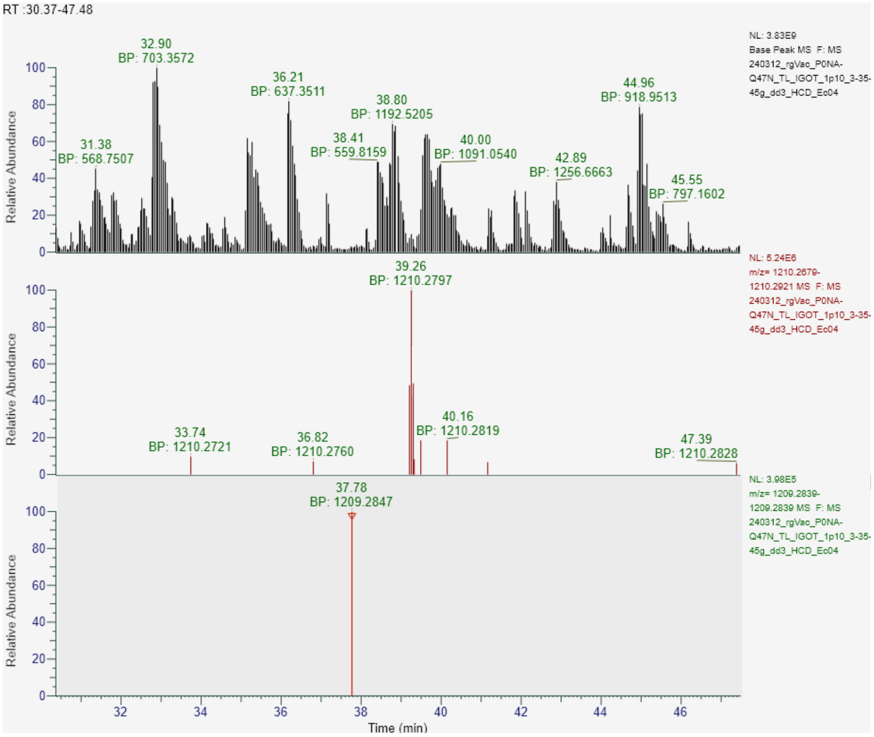

Supplemental Figure 13

Kobayashi *et al*

A Vac2/P0NAΔG-56,57N

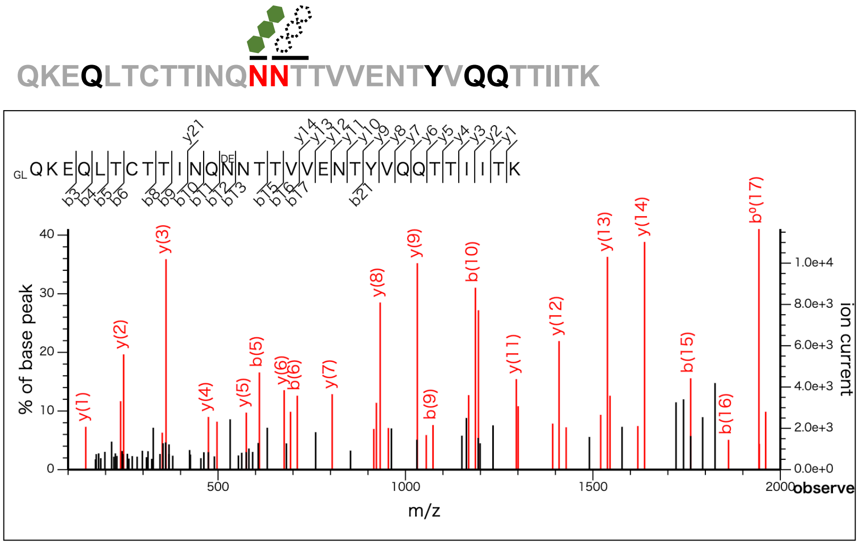

N-glycosylation site was occupied

N-glycosylation sites which are placed in the MS2 fragment could be occupied, while the possibility of modification in the other amino acids could not be denied

MS2 fragment included double NNTT sequons and either of two sites was occupied

N-glycosylation site was not occupied

B

#	b	Seq.	y	#
1	112.0393	Q	—	31
2	240.1343	K	3486.7518	30
3	369.1769	E	3358.6569	29
4	497.2354	Q	3229.6143	28
5	610.3195	L	3101.5557	27
6	711.3672	T	2988.4716	26
7	871.3978	C	2887.4239	25
8	972.4455	T	2727.3933	24
9	1073.4932	T	2626.3456	23
10	1186.5773	I	2525.2979	22
11	1300.6202	N	2412.2139	21
12	1428.6788	Q	2298.1709	20
13	1545.7099	N^DE	2170.1124	19
14	1659.7529	N	2053.0812	18
15	1760.8006	T	1939.0383	17
16	1861.8482	T	1837.9906	16
17	1960.9166	V	1736.9429	15
18	2059.9851	V	1637.8741	14
19	2189.0277	E	1538.8061	13
20	2303.0706	N	1409.7635	12
21	2404.1183	T	1295.7205	11
22	2567.1816	Y	1194.6729	10
23	2666.2500	V	1031.6095	9
24	2794.3086	Q	932.5411	8
25	2922.3672	Q	804.4825	7
26	3023.4148	T	676.4240	6
27	3124.4625	T	575.3763	5
28	3237.5466	I	474.3286	4
29	3350.6306	I	361.2445	3
30	3451.6783	T	248.1605	2
31	—	K	147.1128	1

C

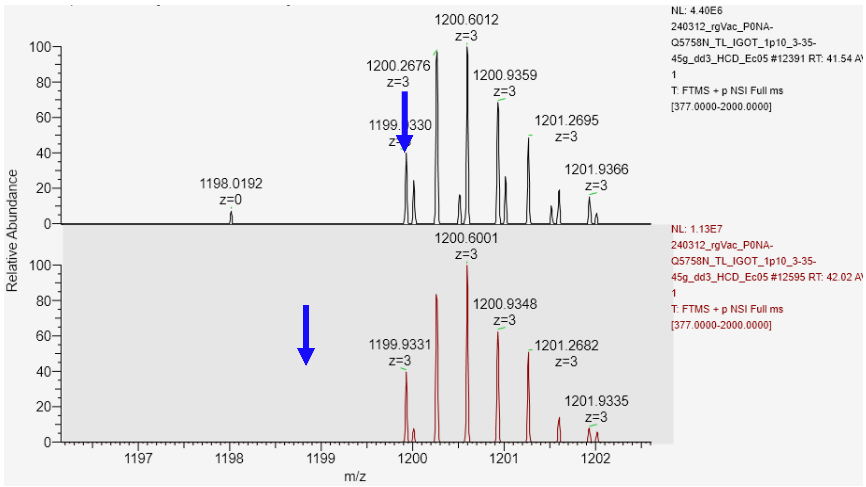

D

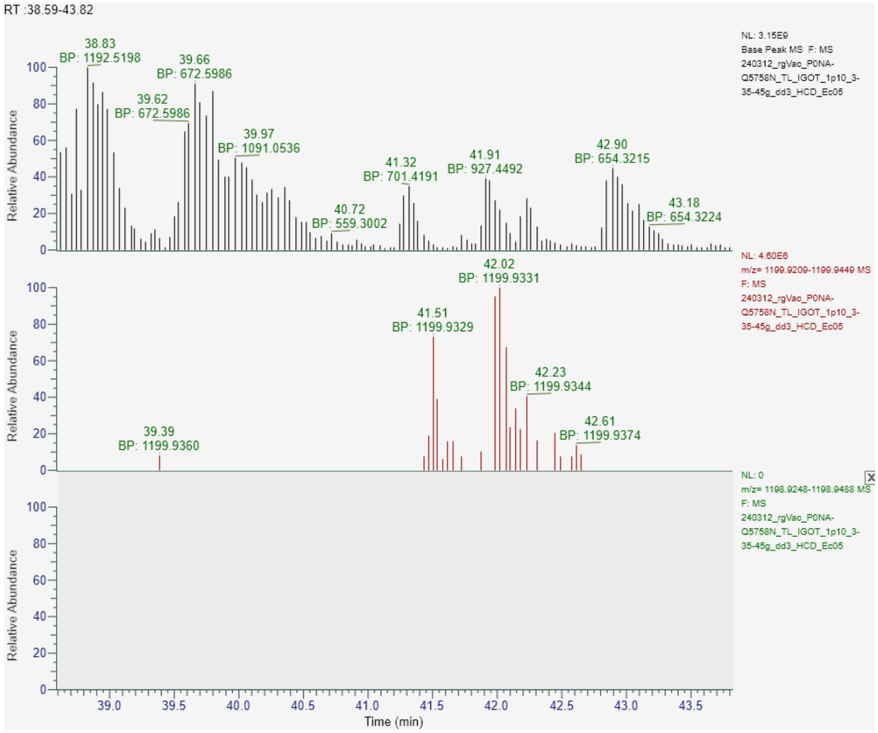

A

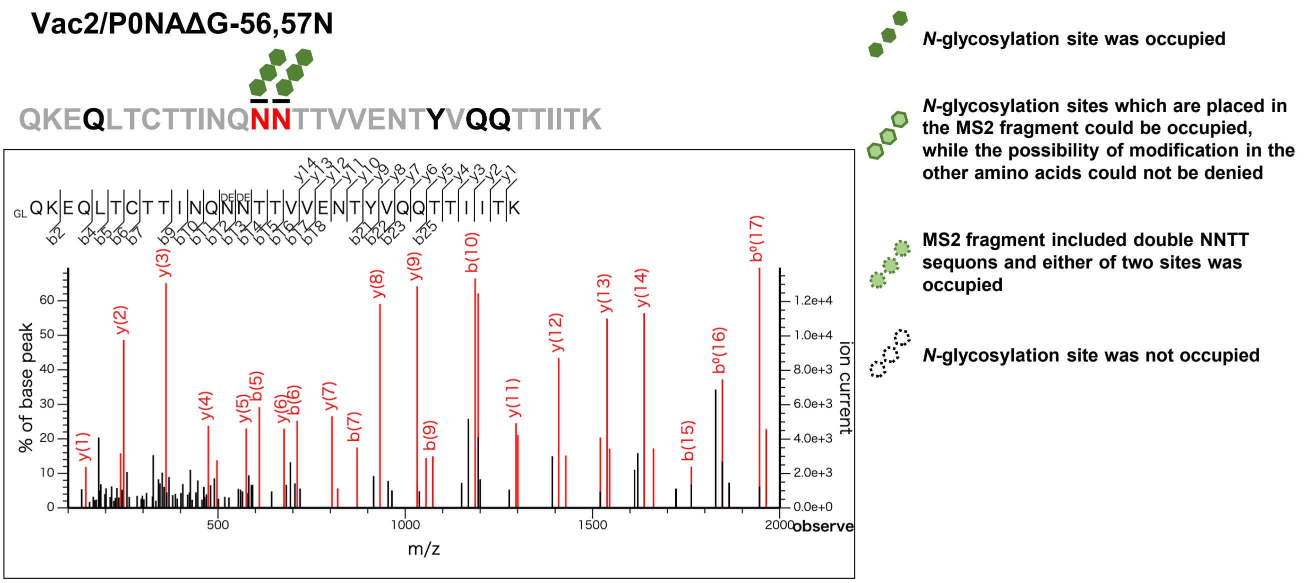

B

#	b	Seq.	y	#
1	112.0393	Q	—	31
2	240.1343	K	3489.7401	30
3	369.1769	E	3361.6451	29
4	497.2354	Q	3232.6025	28
5	610.3195	L	3104.5439	27
6	711.3672	T	2991.4599	26
7	871.3978	C	2890.4122	25
8	972.4455	T	2730.3816	24
9	1073.4932	T	2629.3339	23
10	1186.5773	I	2528.2862	22
11	1300.6202	N	2415.2021	21
12	1428.6788	Q	2301.1592	20
13	1545.7099	N^{DE}	2173.1006	19
14	1662.7411	N^{DE}	2056.0694	18
15	1763.7888	T	1939.0383	17
16	1864.8365	T	1837.9906	16
17	1963.9049	V	1736.9429	15
18	2062.9733	V	1637.8745	14
19	2192.0159	E	1538.8061	13
20	2306.0588	N	1409.7635	12
21	2407.1065	T	1295.7205	11
22	2570.1698	Y	1194.6729	10
23	2669.2383	V	1031.6095	9
24	2797.2968	Q	932.5411	8
25	2925.3554	Q	804.4825	7
26	3026.4031	T	676.4240	6
27	3127.4508	T	575.3763	5
28	3240.5348	I	474.3286	4
29	3353.6189	I	361.2445	3
30	3454.6666	T	248.1605	2
31	—	K	147.1128	1

C

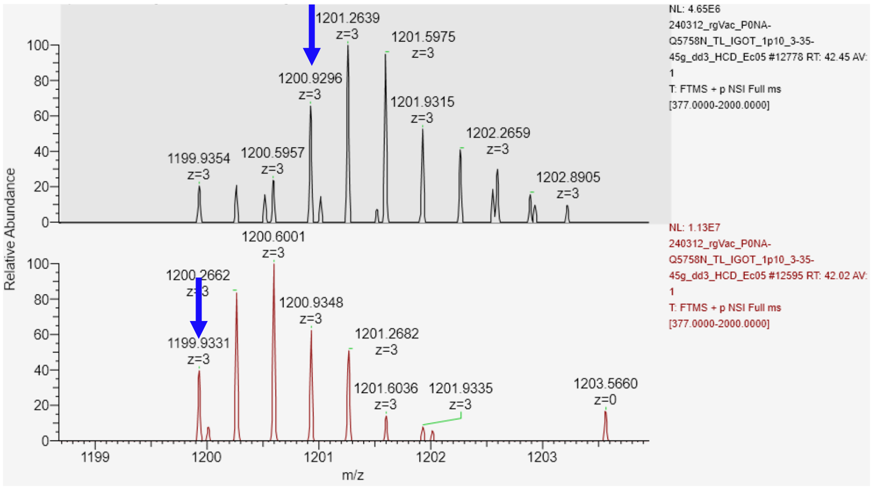

D

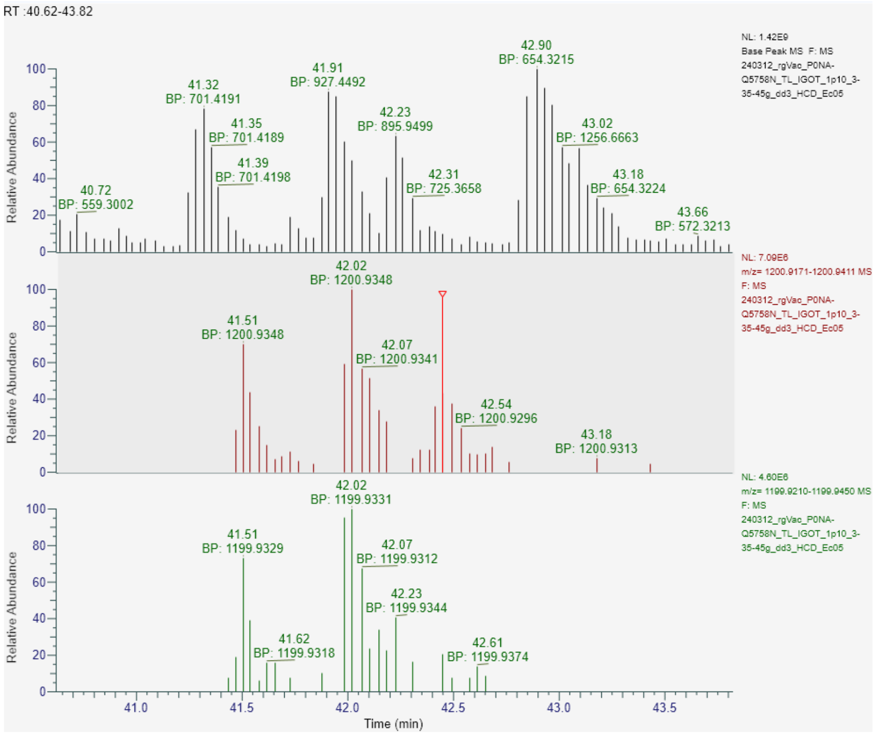

Supplemental Figure 15 **Kobayashi *et al***

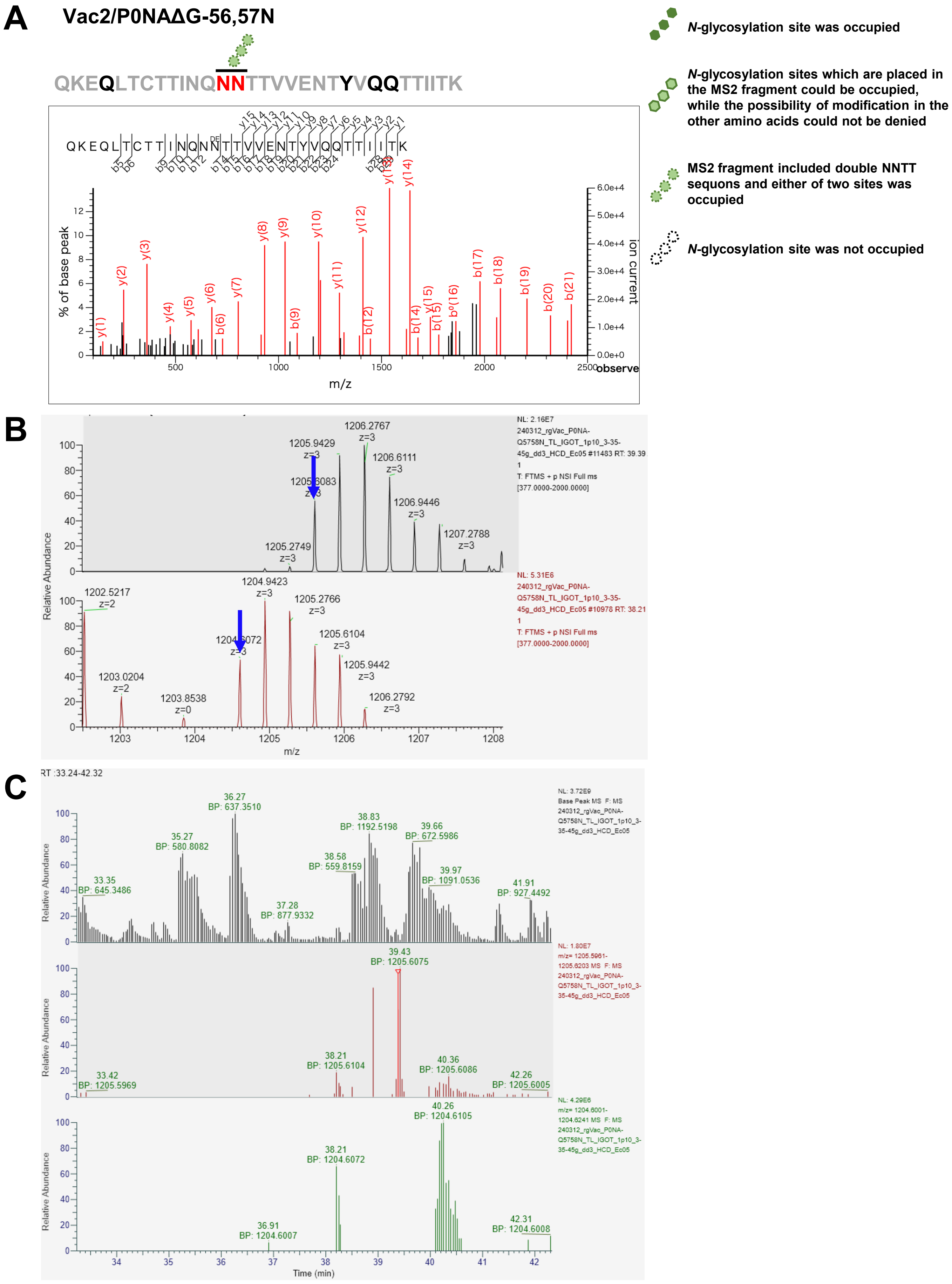

Supplemental Figure 16 Kobayashi *et al*
