# Supplementary material for: Deglycosylation and truncation in the neuraminidase stalk are functionally equivalent in enhancing the pathogenicity of a high pathogenicity avian influenza virus in chickens
Source: J Virol. 2025 Feb 14;99(3):e01478-24. doi: 10.1128/jvi.01478-24 (PMC11915841; doi:10.1128/jvi.01478-24)
Supplement: Table S3 — Amino acid sequences of recovered viruses from six chickens inoculated with L4/P0NAΔG by sanger sequencing. [file jvi.01478-24-s0004.pdf]

**Supplemental Table 3**

Amino acid sequences of recovered viruses from six chickens (#31–36) inoculated with L4/P0NAΔG by sanger sequencing.

| Virus / chicken ID | Clinical course | Organ sample | Amino acid sequence of NA-stalk region* |
|---|---|---|---|
| | | | **30** … **81** |
| L4/P0NAΔG | Original strain | | GLNVSLHLKEKGPKQKEQLTCTTINQQQTTVVENTYVQQTTIITKETDLKTPSY |
| #31 | Euthanized on 4 dpi | Brain | GLNVSLHLKEKGPKQKEQLTCTTINQQQTTVVENTYVQQT------------SY |
| | | Lung | GLNVSLHLKEKGPKQKEQLTCTTINQQQTTVVENTYVQQT------------SY |
| #32 | Seroconversion on 14 dpi | – | No virus rescued |
| #33 | Died on 9 dpi | Brain | GLNVSLHLKEKGPKQKEQLTCTTINQQQTTVVENTHVQQTTIITKETDLKTPSY |
| | | Lung | GLNVSLHLKEKGPKQKEQLTCTTINQQQTTVVENTHVQQTTIITKETDLKTPSY |
| #34 | Euthanized on 5 dpi | Brain | GLNVSLHLKEKGPKQKEQLTCTTINQQQTTVVENTYVQQTTIITKETDLKTPSY<br>GLNVSLHLKEKGPKQKEQLTCTTINQQQTTVVENTHVQQTTIITKETDLKTPSY |
| | | Lung | GLNVSLHLKEKGPKQKEQLTCTTINQQQTTVVENTHVQQTTIITKETDLKTPSY |
| #35 | Died on 4 dpi | Brain | GLNVSLHLKEKGPKQKEQLTCTTINQQQTTVVENTYV------------KTPSY |
| | | Lung | GLNVSLHLKEKGPKQKEQLTCTTINQQQTTVVENTYV------------KTPSY<br>GLNVSLHLKEKGPKQKEQLTCTTINQQQTTVVENTYVQQT------------SY |
| #36 | Euthanized on 4 dpi | Brain | GLNVSLHLKEKGPKQKEQLTCTTINQQQTTVVENTCVQQTTIITKETDLKTPSY |
| | | Lung | GLNVSLHLKEKGPKQKEQLTCTTINQQQTTVVENTCVQQTTIITKETDLKTPSY |

* Dash (-) indicates amino acid deletion and gray color highlights amino acid substitution.

**Kobayashi *et al***
