# Supplementary material for: Deglycosylation and truncation in the neuraminidase stalk are functionally equivalent in enhancing the pathogenicity of a high pathogenicity avian influenza virus in chickens
Source: J Virol. 2025 Feb 14;99(3):e01478-24. doi: 10.1128/jvi.01478-24 (PMC11915841; doi:10.1128/jvi.01478-24)
Supplement: Table S5 — Amino acid sequences of viruses passaged in chicken embryo with L4/P0NAΔG. [file jvi.01478-24-s0006.pdf]

Supplemental Table 5
Amino acid sequences of viruses passaged in chicken embryo with L4/P0NAΔG.

Passaged virus	Amino acid sequence of NA-stalk region*
	**30** … **81**
L4/P0NAΔG	GLNVSLHLKEKGPKQKE<u>QLT</u>CTTINQ<u>QQTT</u>VVENTYV<u>QQTT</u>IITKETDLKTPSY
Embryo Passage 1-1	GLNVSLHLKEKGPKQKE<u>QLT</u>CTTINQ<u>QQTT</u>VVENTYV<u>QQTT</u>IITKETDLKTPSY<br>GLNVSLHLKEKGPKQKEQL-------------ENTYV<u>QQTT</u>IITKETDLKTPSY
Embryo Passage 2-1	GLNVSLHLKEKGPKQKEQL-------------ENTYV<u>QQTT</u>IITKETDLKTPSY
Embryo Passage 3-1	GLNVSLHLKEKGPKQKEQL-------------ENTYV<u>QQTT</u>IITKETDLKTPSY
Embryo Passage 1-2	GLNVSLHLKEKGPKQKE<u>QLT</u>CTTINQ<u>QQTT</u>VVENTYV<u>QQTT</u>IITKETDLKTPSY
Embryo Passage 2-2	GLNVSLHLKEKGPKQKE<u>QLT</u>CTTINQ<u>QQTT</u>VVENTYV<u>QQTT</u>IITKETDLKTPSY
Embryo Passage 3-2	GLNVSLHLKEKGPKQKE<u>QLT</u>CTTTNQ<u>QQTT</u>VVENTYV<u>QQTT</u>IITKETDLKTPSY

* Dash (-) indicates amino acid deletion and gray color highlights amino acid substitution.

Kobayashi *et al*
